# Mutual inhibition model of pattern formation: The role of Wnt-Dickkopf interactions in driving *Hydra* body axis formation

**DOI:** 10.1371/journal.pcbi.1014416

**Published:** 2026-07-13

**Authors:** Moritz Mercker, Alexey Kazarnikov, Anja Tursch, Thomas Richter, Suat Özbek, Thomas Holstein, Anna Marciniak-Czochra

**Affiliations:** 1 Institute for Mathematics and Interdisciplinary Center of Scientific Computing (IWR), Heidelberg University, Heidelberg, Germany; 2 Centre for Organismal Studies (COS), Heidelberg University, Heidelberg, Germany; 3 Institute of Analysis and Numerics, University Magdeburg, Magdeburg, Germany; Weizmann Institute of Science, ISRAEL

## Abstract

The antagonistic interplay between canonical Wnt signalling and Dickkopf (Dkk) proteins is fundamental to tissue organisation, including stem cell differentiation and body-axis formation. Disruptions in this interaction are linked to various human diseases, yet the mechanisms by which β-catenin/Wnt–Dkk interactions give rise to robust spatial patterning remain unclear. A key model system for Wnt-driven pattern formation is the pre-bilaterian organism *Hydra*, where two ancestral Dkk proteins interact with Wnt signalling to self-organise the body axis. While *Hydra* patterning has been extensively studied within the activator–inhibitor framework, a model that directly integrates experimentally identified molecular components has been lacking. Here, we introduce a mathematical model incorporating both Dkk molecules and their experimentally established interactions with Wnt signalling. Numerical simulations and analytical results show that the Wnt–Dkk network alone is sufficient to drive *de novo* body-axis formation across a broad parameter range. The model provides a biologically grounded realisation of the general local activation–long-range inhibition (LALI) principle, in which effective local activation emerges from mutual inhibition rather than molecular self-activation. In contrast to previous *Hydra* models, it explicitly links experimentally characterised Wnt–Dkk interactions to pattern formation, accounts for the experimentally observed role of injury-induced activation, and exhibits robust behaviour under perturbations.

## Author summary

Understanding how organisms form and regenerate complex body structures is a fundamental question in biology. In the freshwater animal *Hydra*, which can regenerate its entire body from a small tissue fragment, a molecular signalling system involving Wnt proteins and their inhibitors, the Dickkopf (Dkk) family, plays a central role in organising the body axis. While these molecules are known to interact, how they collectively generate large-scale spatial patterns has remained unclear, especially since their activity does not fully align with established pattern formation models. In this study, we develop a mathematical model that integrates experimentally observed interactions between Wnt signalling and two Dkk molecules in *Hydra*. The model provides a biologically grounded realisation of the general local activation–long-range inhibition principle through mutual inhibition between molecular subsystems. We show that this interaction architecture is sufficient to explain the emergence of a stable body axis, the requirement of injury–induced activation for regeneration, and the outcomes of experimental perturbations. Our results provide a mechanistic explanation of how Wnt–Dkk interactions can self-organise robust spatial patterns in regenerating tissue, and highlight that classical pattern formation principles can be implemented by different molecular network architectures.

## Introduction

Cnidarians, with their simple body plans and remarkable regenerative abilities, offer a powerful model system for studying fundamental and broadly applicable principles of development and pattern formation [1–3]. Among them, *Hydra* has served as a classic organism in developmental biology for nearly 300 years, owing to its continuous morphogenetic activity, capacity for whole-body regeneration, and amenability to experimental manipulation [4–7]. Body axis formation in *Hydra* is a striking example of a self-organising process: even when dissociated into individual cells, aggregates can regenerate into functional polyps [8,9]. This regeneration showcases *de novo* pattern formation, where cells determine their fate based on positional cues rather than retaining memory of their axial origin [10,11].

To explain such processes, various mathematical models have been proposed. Many adopt a top-down, abstract approach to infer the interactions that could underlie observed patterns. A foundational concept comes from Alan Turing’s theory of reaction–diffusion systems, in which nonlinear reaction kinetics coupled to spatial transport can destabilise an initially homogeneous state and generate spatial patterns. Building on this framework, Gierer and Meinhardt developed an activator–inhibitor reaction–diffusion model for *Hydra* that reproduces key features of pattern formation, such as symmetry breaking and head regeneration, and demonstrates how local self-enhancement of an activator, coupled with long-range inhibition, can account for tissue patterning [12–14]. A later refinement introduced the concept of a slowly changing source density (SD), representing a memory of the body axis that stabilises and aligns spatial domains [15,16].

From a molecular perspective, canonical Wnt/β-catenin signalling governs posterior identity in many organisms, while inhibitors such as Dickkopf (Dkk) proteins define anterior fates by antagonising Wnt activity [17–21]. In vertebrates, this antagonistic Wnt–Dkk interaction plays a central role in axial and head development, and its dysregulation has been linked to a range of human diseases, including cancer and neurodegeneration [18,22]. A comparable mechanism operates in *Hydra* and appears to underlie axis and head formation during regeneration [4,5,23–27]. Nuclear β-catenin and expression of genes like *HyWnt3* mark the oral pole, while *HyDkk1/2/4-A* and *HyDkk1/2/4-C* are expressed in the body column and suppress Wnt signalling downstream [26,27]. These genes are considered evolutionary precursors of vertebrate Dkk homologues [26].

While the activator–inhibitor model provides a compelling theoretical framework for *de novo* pattern formation, its correspondence with known molecular pathways in *Hydra* remains unclear. Although canonical *HyWnt* signalling is a plausible candidate for the activator, a corresponding diffusible inhibitor that fits the model’s assumptions has yet to be identified. Moreover, classical activator-inhibitor models typically describe convergence to a patterned state from any positive initial condition. In contrast, experimental evidence identifies conditions under which proper pattern formation in *Hydra* fails in the absence of a sufficiently strong activation signal. Injury induces a strong and transient activation response, e.g., when head and/or foot are removed, characterised by rapid Ca^2+^/ROS signalling, MAPK activation, and early Wnt transcription at both poles, followed by tissue context-dependent organiser formation [16,28]. In the absence of an open injury, for example, after head removal by hair ligation that preserves epithelial integrity, MAPK activation is reduced and re-patterning is impaired [16,26]. These findings question whether regeneration in *Hydra* can arise solely from amplification of infinitesimal fluctuations in an otherwise homogeneous field, as assumed in classical activator–inhibitor models. Instead, organiser formation requires a sufficiently strong initial activation signal that does not itself impose a positional prepattern.

In addition, *HyDkk* expression patterns do not match the predictions of activator–inhibitor models, which exhibit overlapping maxima of activator and inhibitor at the organiser region [12]. Neither *HyDkk1/2/4-A* nor *HyDkk1/2/4-C* is expressed in the head region, while *HyDkk1/2/4-C* expression additionally decreases towards the aboral end [26,27]. This discrepancy led to Dkk molecules not being considered part of the self-organised pattern formation framework in *Hydra* [14,29], motivating experimental searches for a missing inhibitor to fit the activator–inhibitor model, such as the transcription factor *Sp5*. However, *Sp5* does not diffuse and likewise fails to match the predicted spatial expression profiles [30]. Other candidates, such as thrombospondin (*TSP*) and the secreted protease *HAS-7*, have also been proposed as Wnt inhibitors [31,32], but their expression patterns and functional roles do not conform to the spatial dynamics required by the classical model.

In light of these challenges, it is natural to ask whether one should seek a molecular correspondence with activator–inhibitor models. Instead, we adopt a model-based approach to test whether the experimentally characterised interactions between HyDkk1/2/4-A, HyDkk1/2/4-C, and Wnt/β-catenin signalling are sufficient to account for spatial patterning. In this framework, model variables represent effective activities rather than individual molecular species, with the diffusible Wnt-related component capturing the spatial propagation of secreted Wnt signals and downstream signalling activity. While Wnt secretion and extracellular transport are experimentally established, their quantitative propagation properties in *Hydra* remain insufficiently characterised.

The remainder of this paper is structured as follows. In the Results section, we introduce the mutual inhibition (MI) model, which realises a local activation–long-range inhibition (LALI) system through a mutual inhibition mechanism between Wnt and Dkk components. In this framework, local activation arises via inhibition of an inhibitor, while long-range inhibition is mediated by diffusible components of the Wnt–Dkk network. We present the model formulation, including biological justification and mathematical structure, followed by a comparison with experimental data and simulations of classical and novel perturbation scenarios. Next, we perform an in-depth mechanistic analysis for a reduced one-dimensional version of the model, using both numerical and analytical techniques to investigate conditions for pattern formation, including bistability and Turing instability. We explore the model’s robustness to parameter variations and qualitative modifications. Technical details, extended model variants, and supporting analyses are provided in S1 Appendix.

## Methods and models

**Fig 1:**
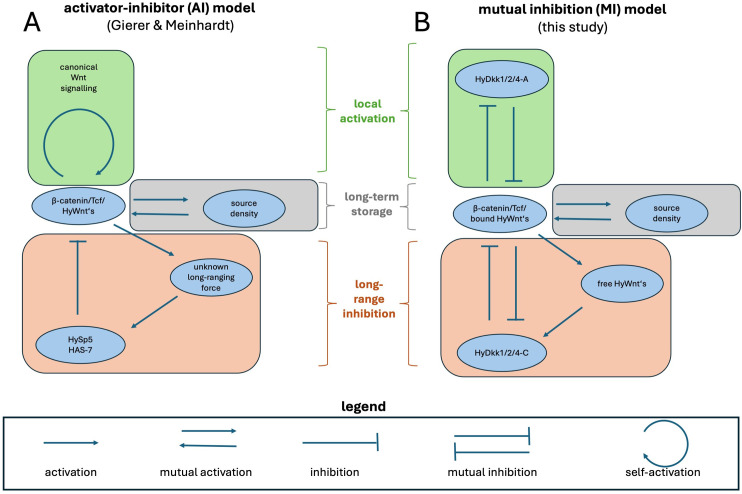
Comparison of the activator-inhibitor (AI) model and the mutual inhibition (MI) model. **(A)** Classical activator–inhibitor model according to Gierer and Meinhardt. Local self-activation is realised by autocatalytic canonical Wnt signalling, coupled to a long-range inhibitory component. The identity of the long-range inhibitor remains unspecified in the original formulation. **(B)** Mutual inhibition (MI) model proposed in this study. Local activation emerges through reciprocal inhibition between canonical Wnt activity (β-catenin/Tcf-bound HyWnt signalling) and HyDkk1/2/4-A. Long-range inhibition is mediated by diffusible Wnt ligands (free HyWnts), which induce HyDkk1/2/4-C that in turn inhibits canonical Wnt activity. The source density represents a slow, long-term positional memory field that mutually interacts with canonical Wnt signalling. Vertical grouping into ‘local activation’ (green shaded boxes), ‘long-term storage’ (grey shaded boxes), and ‘long-range inhibition’ (red shaded boxes) reflects functional modules of the models and does not indicate spatial localisation within the animal. Graphical conventions (activation, inhibition, mutual regulation, and self-activation) are specified in the legend below the figure.

To explore pattern-forming ability of the Wnt-Dkk signalling system, we propose a mechanistic model describing interactions of β-catenin/Wnt and the two Dkk-molecules HyDkk1/2/4-A and HyDkk1/2/4-C in *Hydra*. An overview of the system components and their interactions derived from various experiments is given in Fig 1. The classical activator–inhibitor model is included as a conceptual reference illustrating the LALI principle.

The model is termed the *mutual inhibition (MI) model* because pattern formation arises from two coupled inhibitory feedback loops. First, a local mutual inhibition loop operates between canonical Wnt/β-catenin activity ([β]) and HyDkk1/2/4-A ([*A*]): [β] represses [*A*] expression, while [*A*] inhibits [β] activity. This reciprocal inhibition yields effective local self-activation via inhibition of an inhibitor, without requiring direct autocatalysis of [β]. Second, a spatially extended inhibitory loop involves diffusible Wnt-related activity ([*W*]) and HyDkk1/2/4-C ([*C*]): [β] promotes production of [*W*], [*W*] induces [*C*], and [*C*] in turn inhibits [β], thereby providing long-range inhibition.

The aim of our approach is to determine whether a specific structure of the Wnt–Dkk signalling network is sufficient to explain body axis formation in *Hydra*. The presence of two Dkk components is structurally required in this framework: HyDkk1/2/4-A acts as a short-range inhibitor within the local mutual inhibition loop, whereas HyDkk1/2/4-C is induced downstream of diffusible Wnt activity and mediates spatially extended inhibition (cf., Fig 1). A single Dkk component would not support both local activation via inhibition of a short-range antagonist and long-range stabilisation via a spatially coupled inhibitory field. The choice of the model components and their interactions is motivated by their experimentally documented function [23–27] (cf., below and S1 Appendix). Together, these interactions realise a LALI-type pattern-forming mechanism.

### Model equations of the signalling system and their biological justification

The core of the model is given by reaction–diffusion-type equations describing the dynamics of five components, see Eq. (1)–(5) and Table 1. An overview of model parameters is given in Table 2. The model variable [β] represents the cell-local head-related molecules, e.g., reflected by the patterns of β*-catenin, HyWnt3, HyWnt9/10c,* and *Tcf* expression [23–25]. The model variable [*W*] represents the diffusible Wnt ligands (such as ligands of HyWnt3 or HyWnt9/10c) [33]. Variables [*A*] and [*C*] describe HyDkk1/2/4-A and HyDkk1/2/4-C, respectively [26,27]. The model also incorporates the so-called source density (SD – variable [*S*]), a long-term store of information about the body axis gradient. Although its molecular identity remains unknown, its presence has been demonstrated by multiple experimental studies, see Refs. [4,9,12,34–37] and more details below. Our model does not represent individual molecular species, but effective signalling activities of groups of molecules acting at similar spatial and functional levels. In particular, the variables [β] and [*W*] summarise intracellular canonical Wnt/β-catenin activity and its diffusible extracellular components, respectively. Consequently, the model captures large-scale regulatory organisation rather than detailed gene-specific expression patterns. This abstraction reflects the high molecular redundancy and complexity of the *Hydra* pattern formation system and intends to capture system-level dynamics without explicitly resolving individual transcriptional or translational processes. The model equations read


$$\begin{array}{c} \partial_{t}[\beta ]=\frac{b_{1}[S]}{\left(1+k_{1}[A])(1+k_{2}[C])(1+k_{3}[\beta ]\right)}-c_{1}[\beta ] \end{array}$$ (1)

$$\begin{array}{c} \partial_{t}\;[A]=a_{2}\Delta^{\Gamma }[A]+\frac{b_{2}}{\left(1+k_{4}[\beta ]\right)}-c_{2}[A] \end{array}$$ (2)

$$\begin{array}{c} \partial_{t}[W]=a_{3}\Delta^{\Gamma }[W]+b_{3}[\beta ][S]-c_{3}[W] \end{array}$$ (3)

$$\begin{array}{c} \partial_{t}[C]=a_{4}\Delta^{\Gamma }[C]+\frac{b_{4}[W]}{\left(1+k_{5}[\beta ]\right)}-c_{4}[C] \end{array}$$ (4)

$$\begin{array}{c} \partial_{t}[S]=a_{5}\Delta^{\Gamma }[S]+b_{5}[\beta ]-c_{5}[S], \end{array}$$ (5)

where $\Delta^{\Gamma }[.]$ is the Laplace-Beltrami operator. The model is defined on a surface shell representing the *Hydra* tissue, and for the model analysis we additionally consider a reduced one-dimensional domain. The choice of the domain is discussed below. It should be noted that the model is not formulated as a mass-conserving two- or multi-compartment system. For example, the term $b_{3}[\beta ][S]$ in Eq. (3) represents the activation of diffusible Wnt-related activity by the local Wnt field and the source density, rather than a literal transfer of material. Accordingly, no corresponding influx term appears in Eq. (1), as [β] acts as a regulatory source rather than a mass reservoir. Thus, these formulations capture functional couplings between locally produced and diffusible components without implying mass conservation.

**Table 1:** Model variables and their biological meanings.

Variable name	Explanation
[β]	cell-local head-related molecule complex comprising β-catenin and canonical Wnts, e.g., HyWnt3 and HyWnt9/10c (e.g., Ref. [23–25]).
[*A*]	Dickkopf1/2/4-A - a Wnt antagonist in *Hydra* [26,27].
[*W*]	free (secreted/diffusing) canonical HyWnts
[*C*]	Dickkopf1/2/4-C - a Wnt antagonist in *Hydra* [27].
[*S*]	source density (SD) (also called, e.g., *positional value* or *head forming competence*) - long-lasting information on the body-axis gradient; experimental evidence is provided in [14,24,32,34,39,40] but molecular/physical nature still unknown.

**Table 2:** Overview of the model parameter classes governing *de novo* body axis formation in the mutual inhibition model. Column 3 refers to full (pseudo-3D) simulations reproducing experimental perturbations shown in Figs 2 and 3, based on a single baseline parameter set (see S1 Appendix). Experimental perturbations are implemented either via changes in initial conditions and/or geometry (e.g., transplantation, ALP treatment, head removal, aggregates) or, for molecular perturbations (HyDkk knockdown), by modifying selected production or degradation parameters while keeping diffusion and coupling fixed. Robustness analyses (full pseudo-3D and reduced 1D models) systematically vary parameters around the baseline and are not included in column 3. Parameters related to downstream tentacle and foot patterning are omitted, as these do not affect primary axis establishment (see Discussion).

Parameter class (role)	Parameters	Deviation from baseline (pseudo-3D simulations)
Diffusion (spatial coupling; pattern wavelength selection)	$a_{i}$	Kept constant in all simulations shown in Figs 2 and 3
Production and degradation (activation/inhibition; temporal scaling)	$b_{i}$, $c_{i}$	Modified only for molecular perturbations (HyDkk removal, Fig 3A; Dkk knockdown, Fig 3D)
Inhibition and coupling (nonlinear feedback enabling pattern formation)	$k_{i}$	Kept constant in all simulations shown in Figs 2 and 3
Initial conditions (spatial distribution and abundance of model variables at *t* = 0)	Initial values of model variables	Modified for transplantation/grafting (Fig 2G), ALP treatment (Fig 3B–3D), head removal/regeneration (Fig 2A–2E and 2G), and aggregates (Fig 2D and 2E)
Geometry and mechanics (pseudo-3D tissue representation)	Parameters of tissue geometry and Helfrich energy	Only size of domain changes in simulations of aggregates (Fig 2D–2E)
1D reduced model (effective non-dimensional parameter combinations)	$\zeta_{i}$, $\nu_{i}$, $\tau_{i}$	Not used in pseudo-3D simulations (derived from baseline for the model reduction presented in S1 Appendix)

#### Cell-local β-catenin/Wnt dynamics.

Eq. (1) describes the dynamics of the cell-local canonical Wnt activity [β], representing intracellular β-catenin/Tcf signalling and expression of canonical *HyWnt* genes. The production term is promoted by the source density ([*S*]). The first two inhibitory denominators capture repression by HyDkk1/2/4-A and HyDkk1/2/4-C, consistent with experimental evidence for mutual inhibition between Wnt and Dkk expression [26,27]. The third denominator represents local self-limiting saturation, reflecting constraints on gene expression and protein synthesis, while the final term accounts for degradation of [β]. Additional experimental support is provided in [23–25,33,38,39].

#### Dkk1/2/4-A dynamics.

Eq. (2) describes the dynamics of HyDkk1/2/4-A ([*A*]), an inhibitor of canonical Wnt signalling. The first term represents weak diffusion of [*A*] along the tissue surface. The production term includes basal expression repressed by local Wnt activity ([β]), consistent with experimental evidence for mutual inhibition between HyDkk1/2/4-A and canonical Wnt signalling [26,27]. The final term accounts for degradation of [*A*]. Supporting evidence is given in [26,27,32].

#### Diffusible Wnt dynamics.

Eq. (3) describes the dynamics of diffusible Wnt activity [*W*], representing the extracellular pool of secreted Wnt ligands acting over longer distances. The first term accounts for diffusion along the tissue surface. Production of [*W*] depends on both the source density ([*S*]) and local Wnt activity ([β]), where [*S*] captures permissive conditions arising from slower regulatory layers (such as epigenetic states, chromatin accessibility, or other long-term determinants of cellular identity), and [β] provides the transcriptional drive. The final term accounts for degradation of [*W*]. Experimental support is provided in [23,25,33,38,39]. While the quantitative propagation properties of Wnt in *Hydra* remain experimentally unconstrained, assuming moderate diffusivity provides a plausible approximation; the resulting patterns are robust to variations in the diffusion strength (Fig G in S1 Appendix, panels C–D).

#### Dkk1/2/4-C dynamics.

Eq. (4) describes the dynamics of HyDkk1/2/4-C ([*C*]), a second Wnt antagonist with distinct regulation compared to [*A*]. The first term represents weak diffusion along the tissue surface. Production of [*C*] is positively regulated by diffusible Wnt activity ([*W*]) and repressed by local Wnt activity ([β]), consistent with experimental observations showing upregulation in regions exposed to secreted Wnt and downregulation in the head region where canonical Wnt/β-catenin activity is high [27]. The final term accounts for degradation of [*C*]. For experimental evidence we refer to [27,32].

#### Source density dynamics.

Eq. (5) describes the dynamics of the source density ([*S*]), encoding long-term positional information along the oral–aboral axis of *Hydra*. The first term represents slow diffusion, modelling gradual redistribution through local cell interactions. Production of [*S*] depends on local Wnt activity ([β]), reflecting the experimentally observed induction of long-lasting tissue competence by sustained β-catenin/Tcf signalling [16]. The decay term captures the gradual relaxation of positional information over several days, consistent with regeneration and grafting experiments [24,34,40]. Conceptually, [*S*] represents a coarse-grained positional memory that determines whether a region is permissive for organiser formation. Accordingly, [*S*] evolves on a slower timescale than the Wnt–Dkk signalling system. Experimental evidence is provided in [14,24,32,34,39,40]; further details are given in S1 Appendix.

### Model of the *Hydra* tissue

As the focus of this work is on the ability of the Wnt–Dkk signalling system to control stable body axis formation, we investigate the proposed model both on a simplified one-dimensional domain representing the body axis (’1D model’) and in a more realistic geometry of the *Hydra* tissue (referred to as the ‘pseudo-3D model’), where the tissue is modelled as a deforming two-dimensional surface embedded in three-dimensional space rather than as a fully three-dimensional bulk domain. For the latter, we adopt a mechano-chemical modelling framework coupling the signalling system to a model of an infinitely thin deforming tissue [41–43]. In the pseudo-3D model, the tissue surface is represented as an elastic shell governed by a Helfrich-type bending energy, with the local spontaneous curvature depending on the chemical fields (mainly [β] and [*S*]). This coupling allows chemical patterning to influence the evolving tissue shape. Based on minimisation of the free energy describing elastic tissue deformations, the model yields a fourth-order partial differential equation governing tissue evolution by the gene expression patterns resulting from the MI model. A detailed description of the physical assumptions, initial conditions, and chemo–geometrical coupling is provided in S1 Appendix (section “Mathematical framework for modelling pseudo-3D geometry”). In contrast to the fully coupled mechano-chemical models of [42–44], the current model does not account for any feedback from the mechanical properties of the tissue to the gene expression processes. Consequently, the pattern formation process is induced solely by the chemical signalling system. The purpose of including a realistic geometry in the evolving domain was to examine the potential impact of the underlying geometry on the sensitivity of pattern formation dynamics. To facilitate model analysis, we simplified the system to a one-dimensional domain with zero-flux boundary conditions, which serves as a simplified representation of the *Hydra* body column. The reduction in complexity enabled the efficient execution of numerical simulations and facilitated a more tractable analysis of the pattern formation mechanism.

### Model extensions to account for foot and tentacle dynamics

**Fig 2:**
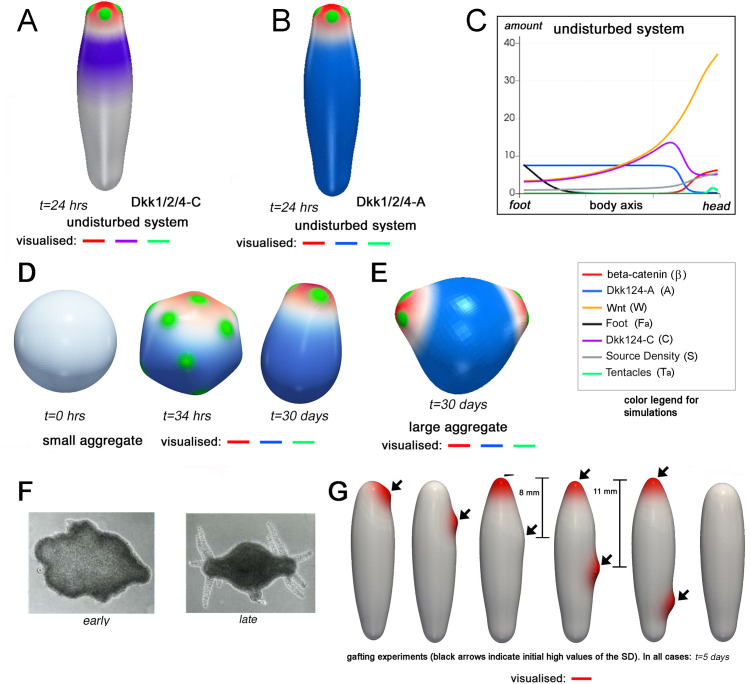
Experiments and simulations of HyWnt–Dkk interactions and resulting patterns. **(A)**-**(B)** Simulated activity fields corresponding to HyDkk1/2/4-C (A) and HyDkk1/2/4-A (B) in the undisturbed polyp after *t* = 24 h. **(C)** Concentration profiles of the different model variables along the body axis in the undisturbed system. **(D)** Different stages of a simulated small aggregate; **(E)** late stage of a simulated large aggregate; **(F)** early and late stages of experimental large aggregates; **(G)** simulated head formation resulting from virtual grafting experiments. Black arrows indicate initially given high values of the SD representing grafts. Colour-scaling is similar in all simulation snapshots in this figure (cf., colour legend). Tentacle and foot structures are included for morphological realism only and do not affect the Wnt–Dkk-driven *de novo* patterning (see Fig I in S1 Appendix).

An additional version of the model includes two separate pattern-formation systems controlling foot and tentacle formation. These processes are not the focus of the present study, as they do not contribute to the pattern formation mechanism of the body axis. Foot and tentacle structures are included solely for visual and biological realism of the simulated *Hydra* morphology and to allow comparison with experimentally observed phenotypes, such as ectopic tentacles after ALP treatment. Both systems are represented by downstream activator–inhibitor modules that do not chemically feed back into the Wnt–Dkk mechanism responsible for axis pattern formation and might influence Wnt/Dkk only, if at all, indirectly via local surface deformations. To test this, we repeated the aggregate simulation shown in Fig 2D with both the foot and tentacle modules removed (Fig I in S1 Appendix). The resulting *de novo* symmetry breaking and final axis pattern remained unchanged, confirming that foot and tentacle formation have no influence on the Wnt–Dkk patterning mechanism. Further details of the foot and tentacle systems, including experimental justification of the model, are provided in S1 Appendix.

#### Tentacle system.

The tentacle subsystem is represented by an activator–inhibitor module that receives positive input from the source density ([*S*]) and negative input from local Wnt activity ([β]). This captures experimental observations that tentacle primordia form preferentially in regions of intermediate head-forming competence, where [*S*] is high but β-catenin/Wnt locally suppresses this system [45]. The resulting field describes the periodic activation of tentacle-specific genes along the upper body column, consistent with observed tentacle spacing and regenerative behaviour.

#### Foot system.

The foot subsystem is modelled analogously as an activator–inhibitor pair regulated by [*S*], but independent of the Wnt–Dkk head system. It represents the basal organiser region, stabilising the aboral pole. The foot activator is enhanced in areas of low [*S*], supporting the formation of a robust basal identity. Both subsystems are mathematically formulated in S1 Appendix.

### Parametrisation strategy

All simulations of the pseudo-3D model shown in the main text were performed using a single baseline parameter set; the corresponding numerical values are listed in Table B in S1 Appendix, and an overview of parameter classes is given in Table 2. Experimental perturbations were implemented relative to this baseline either through changes in initial conditions and/or geometry (e.g., grafting, ALP treatment, head removal, aggregates) or, in the case of molecular perturbations, through modifications of selected production or degradation parameters only. Thus, the simulations do not rely on re-fitting the full parameter set for each experimental scenario.

The parametrisation is consistent with the separation of timescales and spatial ranges in the model. In particular, the source density variable [*S*] evolves on a much slower timescale than the Wnt–Dkk signalling variables, in line with its interpretation as a long-term positional memory field, whereas the diffusible Wnt-related component [*W*] provides the dominant spatial coupling. By contrast, the two Dkk-related components have very small diffusion coefficients, reflecting strongly local effective transport due to binding, uptake, and restricted spread. Using the approximate conversion between numerical and physical units given in S1 Appendix, the baseline diffusion coefficient of [*W*] ($a_{3}=22\times 10^{-3}$) corresponds to an effective transport coefficient of approximately $2.8\times 10^{-3}\;\mu m^{2}/s$. We additionally tested $a_{3}=66\times 10^{-4}$ and $a_{3}=66\times 10^{-2}$, corresponding to approximately $8.4\times 10^{-4}\;\mu m^{2}/s$ and $8.4\times 10^{-2}\;\mu m^{2}/s$, respectively, with only minor qualitative changes in the resulting patterns (cf. Fig G in S1 Appendix, panels C–D). This explored range overlaps with reported effective Wnt transport coefficients of order $5\times 10^{-2}\;\mu m^{2}/s$, while free diffusion may be substantially larger [46].

More generally, robustness analyses in both the full pseudo-3D and reduced 1D settings show that the qualitative patterning behaviour does not depend sensitively on the precise choice of individual parameter values, provided that the key structural requirements of the model are maintained: strong local mutual inhibition, slower source-density dynamics, and sufficiently stronger transport of [*W*] than of the Dkk-related components. In this sense, the model predictions are controlled primarily by parameter relations and timescale/transport hierarchies rather than by fine-tuning of a particular parameter set.

In this context, the model is not intended as a detailed quantitative fit of all underlying molecular processes, but rather as a mechanistically interpretable, phenomenological description. Accordingly, both variables and parameters should be understood as effective quantities describing interactions at the level of signalling activities rather than directly measurable molecular rates. The chosen parameter values aim to be biologically plausible in magnitude and consistent with known qualitative constraints, while capturing the minimal set of interactions required for pattern formation. This approach allows us to assess whether the experimentally supported network structure is sufficient to generate robust spatial patterns without relying on fine-tuning of poorly constrained parameters.

## Results

### Model–experiment comparison and validation

We evaluate the model’s ability to replicate key experiments by comparing simulation outcomes with experimental observations. Most available molecular data on *Hydra* axis formation derive from *in situ* hybridisation, reporting spatial mRNA expression domains. In contrast, the variables in our model represent effective activity fields integrating contributions from multiple molecular components. In this framework, [β] reflects local canonical Wnt/β-catenin activity (as indicated by expression of *HyWnt3*, *HyWnt9/10c*, β-catenin, and *Tcf*), whereas [*W*] represents diffusible Wnt-related activity, which cannot be directly visualised in *Hydra*.

The model is formulated at the level of effective signalling activity and focuses on the slower timescales of axis formation and regeneration (hours to days). Transcriptional regulation, by contrast, operates on faster timescales (minutes) and may exhibit stochastic variability in mRNA levels. While such dynamics can produce heterogeneous granular or ”salt-and-pepper” patterns, our simulation results typically display well-defined expression domains including relatively sharp boundaries. This is consistent with models combining diffusive and non-diffusive variables, where non-diffusive components can exhibit steep gradients or even jump-like transitions [47], whereas diffusive components are spatially smoother. Accordingly, published *in situ* patterns are used as qualitative proxies for the corresponding model variables. This correspondence is expected to be strongest for non- or weakly diffusive components such as [β] and the Dkk molecules, and less direct for diffusible components such as [*W*], for which direct experimental visualisation is currently lacking.

#### MI model reproduces experimentally observed patterns of Dkk expression.

We start with numerical analysis of the wild-type pattern that resembles experimental observations (Fig 2A–2C and Ref. [26,27]). Such a pattern can be established *de novo* if the initial conditions provide a sufficiently strong canonical Wnt signal localised at the head end, which is consistent with head-cutting experiments with a localised signal stemming from the injury. With respect to the resulting stable patterns, the simulations predict the lack of both HyDkk1/2/4-related molecules in the hypostome, showing a sharp expression border beneath the tentacles, with *HyDkk1/2/4-C* expression fading out in the aboral direction (Fig 2A and 2C), and *HyDkk1/2/4-A* strongly expressed in the entire body column (Fig 2B and 2C). The simulated [β] activity field is restricted to the head region, fading within and below the tentacle zone (Fig 2A–2C). Also, the temporal evolution of canonical Wnt activity matches both qualitatively and quantitatively between experimental observations and our simulations (Fig H in S1 Appendix, panel F, vs. Ref. [23]).

The difference in the two HyDkk patterns results from the qualitative differences in the corresponding production terms in the model equations. While HyDkk1/2/4-A is assumed to be constantly produced in the absence of repressing signals, HyDkk1/2/4-C production is modelled downstream of the HyWnt signalling and thus fades out in the aboral direction. To explore the role of the constant production term, we additionally simulated the system with the HyDkk1/2/4-A production depending on SD instead of considering a constant expression. It led to a graded *HyDkk1/2/4-A* expression along the body axis fading out in aboral direction (Fig G in S1 Appendix, panels I–J). In this modified system, the expression of [β] appeared to be distinctly increased in the budding zone (compared to the original model), indicating that body-wide expression of *HyDkk1/2/4-A* might be involved in controlling/suppressing bud formation.

#### MI model reproduces self-organised axis formation in *Hydra* aggregates.

To assess whether the MI model captures genuine *de novo* axis formation, we simulated *Hydra* aggregate experiments. In these experiments, dissociated cells reassemble into initially symmetric tissue spheres without predefined positional information and subsequently undergo spontaneous symmetry breaking to form one or multiple body axes. Accordingly, we initialise the model with a symmetric domain and a random distribution of all biochemical components, including the SD. Small stochastic variations represent intrinsic heterogeneities that provide local competence for Wnt activation. Importantly, these perturbations do not determine organiser position, but act as transient activation events from which stable axes emerge through the intrinsic dynamics of the Wnt–Dkk network. The simulations reproduce *de novo* pattern formation consistent with experimental observations (Fig 2D–2F). Small aggregates develop a single axis (Fig 2D), whereas larger aggregates give rise to multiple heads (Fig 2E), in agreement with experimental data (Fig 2F). Thus, the model captures the experimentally observed scaling behaviour of axis formation, with the number of axes increasing with aggregate size.

#### MI model requires a strong localised signal for head regeneration in cutting experiments.

One of the key challenges for *Hydra* models of head regeneration is the previously demonstrated role of strong localised signalling in cutting experiments. These experiments show that removing the head without creating a wound does not lead to regeneration; only the activation of strong signalling and a localised increase in *Wnt3* expression as part of the wound response enables regeneration [16]. This finding is particularly significant as it directly challenges the classic *de novo* pattern formation mechanism described by activator-inhibitor models. In former theoretical work, we postulated the necessity of bistability in a model to account for such phenomena [48]. The MI model meets these conditions, describing the absence of pattern formation under certain initial conditions, specifically, when an area with high *Wnt3* expression and source density levels is removed, Fig 2G – right hand side. Conversely, the introduction of a strong signal, such as that triggered by the wound response, facilitates pattern formation. A systematic analysis of this mechanism is provided in the next section.

#### MI model reproduces key features of head transplantation and inhibition experiments.

Further, we simulate transplantation experiments (Fig 2G) motivated by the seminal experiments presented in Ref. [34,49,50]. These experiments showed that, under certain conditions, tissue fragments transplanted from one polyp to another can induce a secondary body axis. Our simulations reproduce key features of this behaviour. In particular, head self-inhibition suppresses secondary axis formation when two initiating signals are too close, whereas in the absence of the oral head, grafts can induce a secondary axis at distant positions. Quantitatively, the simulations predict secondary head formation only when the graft is placed more than approximately 50% of the body length away from the oral pole, in agreement with experimental observations [39]. Historical transplantation and fusion-type experiments in *Hydra* further suggested that inhibitory effects of an existing head can act over extended tissue regions and become established over time [51,52]. Motivated by these observations, we additionally simulated serially joined *Hydra* tubes in the one-dimensional model (Fig J in S1 Appendix). In the absence of a terminal head, multiple secondary peaks emerge approximately simultaneously, whereas a pre-existing head at one end suppresses the nearby response such that the more distant secondary peak rises more strongly than the one closer to the existing organiser. In addition, simulations motivated by the tandem ring graft experiments of Ando and Sawada show that repeated pieces of identical axial origin can generate qualitatively different numbers of head-related peaks depending on their original body-axis position (Fig K in S1 Appendix), consistent with the idea that patterning in such extended constructs is governed by the interaction of local activation and long-range inhibition [53]. Thus, the model captures not only distance-dependent suppression of secondary axis formation, but also the temporally developing influence of an existing organiser and the axial-origin dependence of pattern formation in extended tissue constructs.

#### Further *in silico* experiments.

**Fig 3:**
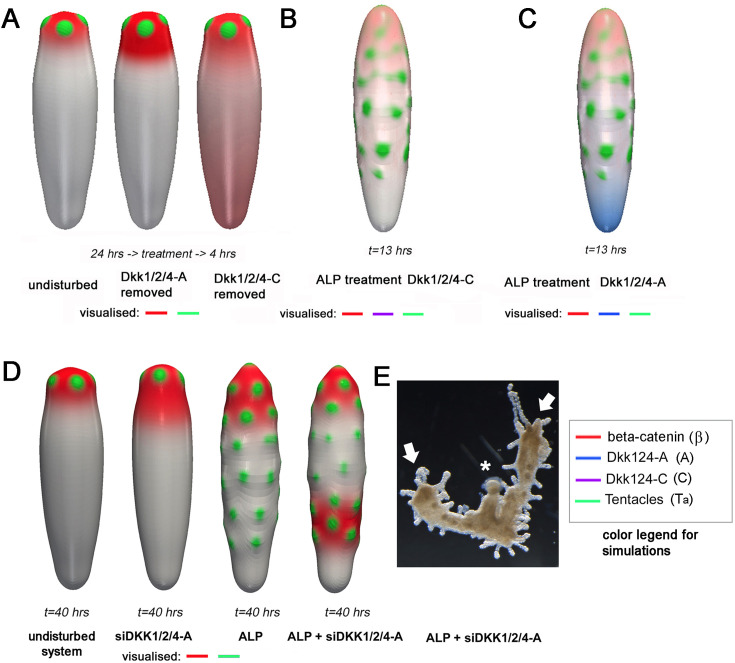
Simulations and experiments of manipulated β-catenin/HyWnt and HyDkk-levels. **(A)** Simulation snapshots of the undisturbed polyp vs. virtual removal of *HyDkk1/2/4-A* vs. *HyDkk1/2/4-C* expression showing the [β] activity field (corresponding to β-catenin/Tcf/bound HyWnt3 domains) and tentacles. **(B–C)** Simulations of *HyDkk1/2/4-A* and *HyDkk1/2/4-C* expression after ALP treatment. **(D)** Simulation snapshots of [β] and tentacle patterns of the undisturbed system vs. different combinations of ALP-treatment and *HyDkk1/2/4-A* knockdown. **(E)** experimental picture of double axis formation after ALP + siDkk1/2/4-A treatment, arrows indicate axes, asterisk represents foot. Colour-scaling is similar in all simulation snapshots in this figure (cf., colour legend).

In the next step, we verify the role of different model components by simulating their manipulated levels and comparing the resulting patterns to experimental data. First, we examine how system behaviour depends on the presence of the two HyDkk1/2/4-related molecules. Specifically, we simulate the undisturbed polyp system until the head pattern is established, as shown in Figs 2A, 2B and 3A (left-hand side). We then perturb the system by virtually removing the expression of one of the two *HyDkks*. In line with experiment, we observe a distinct expansion of [β] in the head region following the removal of HyDkk1/2/4-A (Fig 3A (middle) and Ref. [26]) and body-wide ectopic activation of the [β] field after removing *HyDkk1/2/4-C* (Fig 3A, right-hand side as well as Ref. [27]). The only difference between the simulations and experiments is that [β] production appears relatively diffuse and homogeneous after virtual reduction of *HyDkk1/2/4-C* expression (Fig 3A, third snapshot), whereas experiments report a more granular pattern of *HyWnt3* expression [27]. This discrepancy likely reflects that the experimental patterns correspond to *HyWnt3* mRNA expression, whereas the model variable [β] represents an effective signalling activity integrating multiple molecular components. As a result, the simulated patterns appear spatially smoother than the expression pattern of a single gene.

In addition, we simulate the activation of canonical HyWnt signalling via ALP treatment (Fig 3B and 3C, as well as Fig H in S1 Appendix, panels A–D). Experimentally, both HyDkks are suppressed by this treatment, along with the development of ectopic tentacles along the body column [26,27]. *HyDkk1/2/4-C* expression appears to be more sensitive to β-catenin/HyWnt3 levels, as the reduction in *HyDkk1/2/4-A* expression occurs later than the reduction in *HyDkk1/2/4-C* expression [26,27]. These observations align with our simulation results during ectopic tentacle development (Fig 3B, 3C). However, we note that in later stages of the simulations, Dickkopf patterns re-establish (1D concentration profiles in Fig H in S1 Appendix, panels A–D), suggesting that the model can reproduce the data only as transient patterns. This may suggest that ALP-driven effects are initially present but reversible. Finally, we simulate recent *HyDkk1/2/4-A* knockdown experiments [32] with and without ALP treatment (Fig 3D). Similar to the experimental results (Fig 3E), the combination of ALP treatment and *HyDkk1/2/4-A* knockdown leads to the development of a secondary ectopic axis, marked by an additional region with high activity of the canonical HyWnt signalling field in our simulation results, but again, this is observed as a transient pattern.

### Analysis of the mechanism underlying pattern formation

To determine under which conditions the MI system can exhibit symmetry breaking and pattern formation, we first analyse the structure and stability of spatially homogeneous steady states (cf., S1 Appendix, section ‘Spatially homogeneous steady states and their stability’). Stability of these states implies the absence of patterns in their vicinity, whereas their destabilisation leads to symmetry breaking. In particular, Turing instability (diffusion-driven instability; DDI) arises when two key conditions are met: (i) a sufficient separation of spatial scales due to sufficiently different diffusion coefficients, and (ii) an effective local self-activation mechanism (here realised via mutual inhibition). Under these conditions, small perturbations of a homogeneous state can grow and give rise to spatial patterns. Systems coupling diffusive and non-diffusive components can, additionally, exhibit *far-from-equilibrium* patterns characterised by jump discontinuities [47,54,55]. Such solutions can emerge due to the system’s bistability. While Turing instability may act as a trigger, the emergence of *far-from-equilibrium* patterns can occur independently of the Turing mechanism. Models exhibiting both bistability and DDI have been studied both in full reaction–diffusion systems [56] and in settings with non-diffusing components [47]. For theoretical results on linear and nonlinear stability in reaction–diffusion–ODE models, we refer to [54,57,58].

To better understand the nature of the patterns observed in our simulations, we analyse the stability of branching stationary solutions near the DDI bifurcation point (cf., S1 Appendix, section ‘Semi-analytical approach to the analysis of branching patterns’). Since a rigorous analysis of complex models is often infeasible, we complement it with numerical analysis. Guided by analytical insights, we analyse the model for various fixed parameter values. This is further supported by a sensitivity analysis, which assesses the model’s robustness to parameter and nonlinearity variations and confirms the validity of the results within specific parameter regimes.

#### One-dimensional model reduction.

In this section (with more details given in S1 Appendix, section ‘One-dimensional model reduction’), we focus on a one-dimensional version of the model given by equation (1)–(5) to systematically analyse its dynamics depending on the parameters and initial conditions. Our goal is to provide a deeper understanding of the simulation results discussed earlier. We begin by examining the model’s ability to generate patterns. This involves analysing the mathematical structure of the model, the ability of symmetry breaking and the stability of the emerging patterns.

The reduced model is obtained by approximating the complex domain of *Hydra* by an interval [0,1] and rescaling time and model variables in equations (1)–(5). The technical details of the model reduction, analytical approach, and numerical implementation are provided in S1 Appendix. To compare the reduced system with the original model, we apply a computational fitting procedure using data derived from the pseudo-3D simulation (Fig 2C). These data are obtained by averaging along the axis perpendicular to the body axis, followed by min–max normalisation. Parameter estimates are identified by minimising the least-squares residual between the data profiles and the output of the reduced model. This procedure demonstrates that the reduced model reproduces the same dynamics as the pseudo-3D model, with appropriate parameter adjustments. Further numerical details are included in Table A in S1 Appendix.

#### MI model reveals bistable behaviour in uniform steady-state structures.

**Fig 4:**
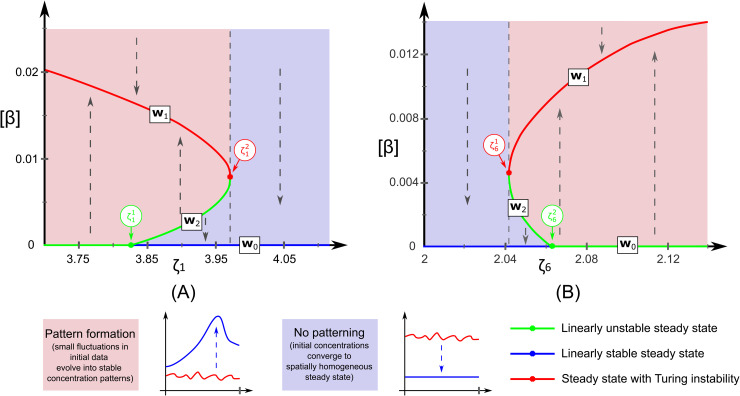
Bifurcation diagram illustrating the qualitative changes in pattern formation in the one-dimensional version of model (1)–(5), induced by variations in the rescaled reaction parameters $\zeta_{1}:=b_{2}/c_{2}$ (effective production rate of [*A*]) and $\zeta_{6}:=b_{1}/(c_{1}c_{5})$ (effective production rate of [β]). The vertical axis represents the first component of the homogeneous steady states. Arrows indicate whether initial conditions converge to the pattern formation regime or decay towards the trivial steady state. Coloured bullets mark the boundaries of the bistable region: $\zeta_{1}^{1}$ and $\zeta_{1}^{2}$ in panel **(A)**; $\zeta_{6}^{1}$ and $\zeta_{6}^{2}$ in panel **(B)**. All other model parameters are set to the values listed in Table A in S1 Appendix.

Analysis of the structure of the spatially uniform steady states demonstrates a bifurcation from a semi-trivial state $\left([\beta ]=0,[A]=\zeta_{1},[W]=0,[C]=0,[S]=0\right)$, where $\zeta_{1}=b_{2}/c_{2}$, resulting in the existence of additional spatially homogeneous solutions and a change of stability, see Fig 4. We identify two complementary bifurcation parameters, $\zeta_{1}:=b_{2}/c_{2}$ and $\zeta_{6}:=b_{1}/(c_{1}c_{5})$, which describe the effective production rates of [*A*] and [β], respectively. More precisely, linear stability analysis of the semi-trivial steady state yields an instability condition, $\zeta_{6}>1+\zeta_{1}$, which marks the bifurcation point and highlights the complementary relationship between the two bifurcation parameters. The corresponding bifurcation diagrams are shown in Fig 4A and 4B. The remaining parameter values are fixed according to the parameter fit to the three-dimensional model discussed above (see Table A in S1 Appendix).

**Fig 5:**
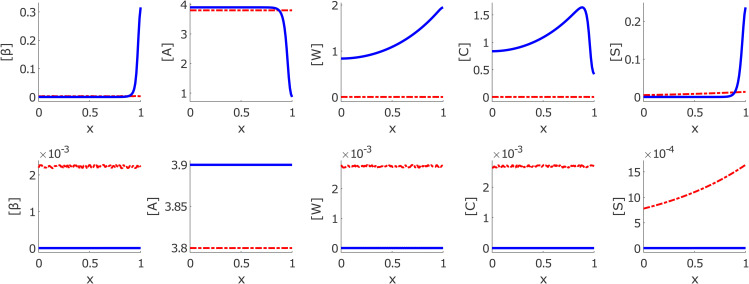
Example of bistable behaviour in the one-dimensional reduced model. Initial data are taken in vicinity of the homogeneous steady state $w_{1}$. When small perturbations lie in the basin of attraction of $w_{1}$, pattern formation occurs and initial data evolve to the steady-state patterns (first row). Otherwise, when the steady state is perturbed in the *direction* of the trivial steady state $w_{0}$, no pattern formation occurs and initial concentrations decay to the trivial steady state (second row). Parameter values used in the simulations are given in Table A in S1 Appendix, except $\zeta_{1}=3.9$. Blue colour shows the final concentration profiles, while red colour denotes the initial concentration values.

The structure of the bifurcation diagram and the stability of different branches of spatially uniform solutions are determined based on the model analysis presented in S1 Appendix (Lemma S1–S4 and Corollary S1) and numerical computations, employed whenever a complete analytical understanding was not feasible. To this end, we investigate the model for varying either $\zeta_{1}$ or $\zeta_{6}$, while fixing all other parameters. As theoretically predicted, for sufficiently small values of $\zeta_{1}$, we observe instability of the semi-trivial stationary solution, denoted by $w_{0}$, and existence of exactly one non-trivial spatially uniform steady state $w_{1}$ which is stable for the system without diffusion. Numerical computation of the system linearised at this positive steady state indicates Turing instability. This stays in agreement with the model simulations showing globally stable pattern formation. Above the critical value (transcritical bifurcation point), the semi-trivial state becomes stable and gives branching to an unstable homogeneous steady state $w_{2}$ to eventually collide with $w_{1}$ for the parameter value corresponding to the saddle-node bifurcation point. This results in a bistable regime for the parameter $\zeta_{1}^{1}<\zeta_{1}<\zeta_{1}^{2}$. This means that for initial concentration values in the basin of attraction of the trivial steady state $w_{0}$, there is no pattern formation, and initial data decay to the trivial steady state $w_{0}$. On the other hand, if initial data lie in the basin of attraction of the steady state $w_{1}$, then we observe the formation of patterns. Here, unstable steady state $w_{2}$ acts like a separation hyperplane between two regimes, see Fig 5. For $\zeta_{1}>\zeta_{1}^{2}$ no pattern formation occurs and the only existing spatially uniform steady state $w_{0}$ becomes a stable attractor. Analogous results are obtained with respect to the bifurcation parameter $\zeta_{6}$, as shown in the bifurcation diagram Fig 4B.

Translated into the language of experimental research, this means that pattern formation or regeneration does not occur for arbitrary perturbations of the initial conditions, but critically depends on the nature and strength of those perturbations. This, in turn, explains why the MI model aligns with the experimental observation that regeneration only occurs when the initial conditions provide a sufficiently strong stimulus through wound signalling.

#### MI model exhibits Turing pattern formation.

Numerical simulations show emergence of stable stationary patterns. To gain analytical insight, we perform a bifurcation analysis near the onset of diffusion-driven instability (see S1 Appendix for details). Since not all expressions can be obtained explicitly, key coefficients are evaluated numerically. We consider two representative parameter regimes–one exhibiting hysteresis (bistability) and one without–and use the dominant diffusion coefficient as the bifurcation parameter. The critical value is computed numerically, and bifurcation theory is then applied to derive asymptotic expressions for the branching stationary solutions and assess their linear stability. In both regimes, the branching solutions are found to be stable, in agreement with direct simulations. While this analysis is not fully rigorous, it provides strong evidence for the existence of Turing patterns in the MI system.

#### MI model is robust with respect to parameter perturbations.

Next, we numerically investigate the robustness of pattern formation with respect to variations in model parameters. To demonstrate this robustness, we use the parameter values listed in Table A in S1 Appendix. We then explore the hypercube in parameter space for rescaled reaction rates $\zeta_{i}$ within a large range $\left[\zeta_{i}/100,100\;\zeta_{i}\right]$, parameters are therefore varied by a factor of 10^4^. To estimate the regions within this hypercube where pattern formation occurs, we employ Markov Chain Monte Carlo (MCMC) methods. The criterion for admissibility is the existence of at least one homogeneous steady state exhibiting Turing instability. For the chosen parameter values, we did not identify any cases where this criterion was not met, indicating the robustness of the observed pattern formation. Of course, the range of parameters studied here did not cross the previously discussed points of transcritical bifurcation, beyond which the only stable equilibrium state of the system would be the semi-trivial state. Another critical parameter is the largest diffusion coefficient in the system, which must be sufficiently large to permit Turing instability.

#### MI model is robust with respect to a range of qualitative changes.

Finally, we assess the robustness of the reduced model (for equations, cf. S1 Appendix) with respect to modifications in the reaction terms. We consider two variations of the model. In the first, the reaction terms are multiplied by their respective denominator expressions. This modification can be interpreted as describing inhibition at the level of degradation or receptor binding, rather than at the level of production. Another system’s perturbation concerns using higher-order terms in Hill functions, i.e., modulating the inhibition process. For the parameter values examined, both variations exhibit the same qualitative pattern formation behaviour as the original one-dimensional model, suggesting its robustness. For further technical details, see S1 Appendix.

## Discussion

The mutual inhibition (MI) model developed in this study provides a mechanistic account of self-organised axis formation in *Hydra*, linking experimentally observed Wnt–Dkk interactions to robust pattern formation. We show that this interaction structure is sufficient to generate stable axes, reproduce regeneration outcomes, and capture the size-dependent scaling behaviour observed in aggregates. In this way, the model realises the general local activation–long-range inhibition (LALI) principle of *de novo* pattern formation within a biologically grounded framework.

In their original work, Gierer and Meinhardt showed that LALI can be implemented by different reaction–diffusion schemes, including both the classical activator–inhibitor system and the activator–depleted–substrate mechanism [8,14]. Later, Meinhardt generalised this concept to multi-component systems, emphasising that activation and inhibition can arise as properties of interacting subsystems (e.g., via mutual inhibition or inhibition of an inhibitor), rather than being tied to individual molecular species [13,14]. In this spirit, the MI model implements the LALI principle through experimentally established antagonistic interactions between Wnt signalling and Dkk molecules in *Hydra*. Local activation emerges from the mutually inhibitory Wnt–Dkk subsystem, which stabilises regions of high Wnt activity, whereas long-range inhibition is mediated by diffusible Wnt-related signals together with Dkk gradients. Although both Dkk molecules are expressed in overlapping domains and participate in mutual inhibition with canonical Wnt signalling, they play distinct functional roles. HyDkk1/2/4-A primarily contributes to the local activation loop, whereas HyDkk1/2/4-C is involved in long-range inhibition (cf. Fig 1 and Fig G in S1 Appendix, panels E–H). This distinction arises because local activation is realised via inhibition of the inhibitor HyDkk1/2/4-A. Thus, HyDkk1/2/4-A does not act as an activator itself, but contributes to a module that functionally realises local activation through inhibition of an inhibitor. Its inhibitory effect on canonical Wnt signalling is therefore essential for pattern formation (Fig H in S1 Appendix, panel E). Thus, the MI model links the abstract LALI framework to specific molecular interactions that account for the observed Wnt/Dkk patterns in *Hydra*. Importantly, mutual inhibition between canonical Wnt signalling and HyDkk1/2/4-C is not required for stable axis formation, as shown by control simulations (Fig G in S1 Appendix, panels E–H), indicating that the overall network architecture is sufficient to realise the LALI mechanism.

The MI model has direct biological implications, highlighting key processes that require further experimental characterisation to fully understand pattern formation in *Hydra*. It further clarifies how interactions between Dkk molecules and canonical Wnt signalling can give rise to spatial patterning in *Hydra* through mutual inhibition. Although Wnt–Dkk interactions are well established in developmental contexts [18,59], their role in spatial pattern formation has remained unclear. Our results suggest that such interactions can constitute a general pattern-forming motif that extends beyond *Hydra*. Importantly, the MI model yields experimentally testable predictions. It predicts distinct functional roles of the two Dkk molecules, with HyDkk1/2/4-A primarily contributing to local activation and HyDkk1/2/4-C to long-range inhibition. It further predicts threshold-like behaviour of regeneration, where successful axis formation requires sufficiently strong initial activation, e.g., during regeneration of extremities, in developing aggregates or after grafting. In particular, injury signals are represented in the MI model as transient activation inputs that push the system beyond a threshold while preserving genuine *de novo* pattern formation. These predictions provide concrete directions for future experimental validation.

A central assumption of the model is the presence of effective transport of Wnt-related signals. While studies in other systems provide evidence for Wnt propagation over relevant spatial scales [46,60,61], direct quantitative characterisation in *Hydra* is still lacking. Accordingly, the diffusion term in the model should be interpreted as a phenomenological representation of multiple possible propagation mechanisms, potentially including the combined spread of several Wnt ligands [33], biomechanical coupling [62], bioelectrical signalling [63], or active transport along cellular protrusions [64].

A limitation of the present study is that it does not explicitly distinguish between body axis formation and head organiser formation, although recent work suggests that these processes may be mechanistically distinct [65]. The MI mechanism primarily operates at the scale of the body axis, whereas organiser formation appears to involve additional regulation at smaller spatial scales. This distinction is consistent with experimental observations indicating differential roles of canonical Wnt components during regeneration. In particular, *HyWnt9/10c* is associated with early activation, while *HyWnt3* is more closely linked to organiser formation [16,33]. Correspondingly, *HyWnt9/10c* knockdown results in complete regeneration failure, whereas *HyWnt3* knockdown still permits tentacle formation, indicating that the body axis remains intact [16]. In addition, the model represents several molecular components, including β-catenin, HyTcf, *HyWnt3*, and *HyWnt9/10c*, as effective variables. While this abstraction enables a tractable system-level description, it also highlights gaps in current understanding. For example, the broader spatial distribution of nuclear β-catenin compared to *Wnt3* expression suggests the presence of additional regulatory mechanisms that restrict local gene expression domains [65]. Future work should address these differences and investigate how Dkk perturbations affect specific components of the Wnt pathway.

Recent studies have highlighted that pattern formation in *Hydra* involves not only biochemical interactions, but also mechanical and cytoskeletal processes. In particular, the supracellular actin cytoskeleton has been shown to form an active nematic field, in which topological defects can act as organising centres during regeneration [66]. These defects are associated with localised mechanical stresses and recurrent rupture events, consistent with a mechanochemical feedback between tissue strain, actin organisation, and morphogen production [67]. Complementary experiments demonstrate that mechanical perturbations can directly influence patterning outcomes. For example, externally induced actin defects can rescue organiser formation in otherwise non-regenerating geometries, while anisotropic stretching biases the orientation of emerging structures in aggregates [68,69]. Earlier work further established that tissue stretching and mechanical oscillations are linked to Wnt activation and head organiser formation [70,71], culminating in the identification of a mechano-chemo-osmotic feedback loop driving *de novo* organiser formation [44]. Together, these findings indicate that biochemical signalling, tissue mechanics, and cytoskeletal self-organisation are tightly coupled processes that may operate on distinct but interacting spatial and temporal scales. In this context, the MI model focuses on biochemical patterning at the scale of the body axis, while mechanochemical processes likely act in a complementary manner to bias, stabilise, or refine organiser formation [65].

Beyond its biological implications, this study highlights a complementary modelling perspective. Classical top-down approaches have been instrumental in identifying general pattern-forming principles, but the increasing number of minimal networks capable of generating Turing-like patterns [72,73] makes it difficult to uniquely relate abstract models to specific molecular systems. Here, we adopt a bottom-up strategy, constructing the model from experimentally characterised components and interactions. This approach does not replace existing theoretical frameworks, but complements them by directly linking molecular interactions to established pattern formation principles. From a theoretical perspective, the MI model belongs to a class of systems that couple diffusive and non-diffusive components. Such reaction–diffusion–ODE systems can exhibit dynamics beyond classical Turing mechanisms [54,74–78], including far-from-equilibrium patterns with sharp spatial transitions [47,55,79]. Our results show that in the presence of multiple diffusive components, stable Turing patterns can coexist with bistability, highlighting a class of models that remains insufficiently explored.

In summary, this study establishes a mechanistic link between experimentally characterised Wnt–Dkk interactions and the general principles of self-organised pattern formation. We show that mutual inhibition is sufficient to realise a LALI-type mechanism, providing a concrete molecular implementation of *de novo* axis formation in *Hydra*. By connecting an experimentally supported interaction network to robust pattern-forming behaviour, the MI model helps bridge the gap between abstract theory and biological systems. Integrating this framework with future experimental and mechanochemical studies will be important for developing a more complete understanding of pattern formation in *Hydra*.

## Supporting information

S1 AppendixTable A. Parameter values of the one-dimensional model.Values of model parameters obtained by fitting the one-dimensional model (S2) to the pattern data obtained from numerical integration of the pseudo-3D model. **Table B. Baseline parameter values used for the pseudo-3D simulations.** Baseline parameter values used for the pseudo-3D simulations, including the mutual inhibition (MI) model and the auxiliary tentacle and foot modules. **Fig A. Intersections of functions *f*_1_(*w*) and *f*_2_(*w*).** Intersections of the functions *f*_1_(*w*) and *f*_2_(*w*) for different values of the model parameters $\zeta_{i}$, i=1,…,6. The value of $\zeta_{1}$ is indicated in each panel, while all other parameters are fixed to the values given in Table A. **Fig B. Comparison of one-dimensional steady-state patterns with pseudo-3D simulation data.** Steady-state pattern obtained from the numerical simulation of the one-dimensional model (S2) with the parameter values from Table A, compared with concentration profiles from the pseudo-3D simulations averaged in the direction perpendicular to the body axis. Both concentration profiles are scaled to the interval [0,1] using min–max normalisation. **Fig C. Examples of pattern formation for different initial conditions.** Three examples of pattern formation in the one-dimensional model. Parameter values used in the simulations are given in Table A. Initial data are taken as small perturbations of the homogeneous steady state. Blue curves show the final concentration profiles, while red curves denote the initial concentration values. **Fig D. Semi-analytical bifurcation analysis of the one-dimensional system.** Results of the semi-analytical bifurcation analysis of the one-dimensional system. (A) Real parts of the eigenvalues of the linear operator $L_{N}(\nu_{cr})$. Real eigenvalues are shown in blue, while the real parts of complex eigenvalues are shown in green. The critical eigenvalue is highlighted in red. (B) Unstable modes as functions of the diffusion coefficient of [*W*]. (C) Asymptotics of the secondary stationary solution for the first component, evaluated at ϵ=0.01 (blue), compared with the numerical solution computed with the same parameter values (red). The green dashed line represents the respective component of the spatially homogeneous steady state *w*_1_. Initial conditions for the simulation are small random perturbations of the stationary state. **Fig E. Pattern of the model variant with feedback in degradation terms.** Example pattern obtained by numerically integrating system (S34)–(S38), in which regulatory feedback is introduced via modified degradation terms. Initial data are taken as small perturbations of the homogeneous steady state *w*_1_. Blue curves show the final concentration profiles, while red curves denote the initial concentration values. **Fig F. Pattern of the model variant with nonlinear reaction terms.** Example pattern obtained by numerically integrating system (S39)–(S43), in which selected reaction terms are replaced by higher-order nonlinear terms. Initial data are taken as small perturbations of the homogeneous steady state *w*_1_. Blue curves show the final concentration profiles, while red curves denote the initial concentration values. **Fig G. Parameter sensitivity analysis and model variants.** (A–D) Stable pseudo-3D patterns and extracted one-dimensional concentration profiles for models with different diffusion rates of the [β] complex (B) or of [*W*] (C–D), compared with the unperturbed system (A). (E–H) Simulated rescaled distribution of Dkk1/2/4-C (E–F) and [β] (G–H) expression for the undisturbed system (E,G) and for a system in which no [β]-based inhibition of Dkk1/2/4-C is included (F,H). In the latter case, the expression patterns resemble the classical activator–inhibitor model. (I–J) Relative distribution of simulated Dkk1/2/4-A (blue), [β] (red), and tentacle (green) expression for the undisturbed system (I) and for a system without constant Dkk1/2/4-A expression but with [*S*]-induced expression instead (J). (K–M) Concentration profiles of the unperturbed system (K) compared with systems with equal Dkk1/2/4-A and Dkk1/2/4-C diffusion rates. In (L), $a_{2}=a_{4}=1\times 10^{-6}$; in (M), $a_{2}=a_{4}=1\times 10^{-7}$. **Fig H. ALP-treatment simulations.** (A–D) Pseudo-3D results and extracted one-dimensional concentration profiles for different time points after simulated ALP treatment. Red colour represents the [β] complex, blue colour Dkk1/2/4-A, and green colour tentacles. (E) Snapshot of a simulation without Dkk1/2/4-A-based inhibition of the [β] complex. The observed patterns, such as [*W*] gradients, result from [*S*] initial conditions only and vanish over time, indicating that the mutual negative feedback loop is required for local self-activation. (F) Simulated temporal development of relative β-catenin expression strength after head removal. **Fig I. Symmetry breaking in aggregate simulations without auxiliary modules.** Different stages of the pseudo-3D simulation of the *Hydra* aggregate system without foot and tentacle modules. The simulation corresponds to Fig 2D in the main text but excludes both auxiliary pattern-formation subsystems. The resulting de novo symmetry breaking and Wnt–Dkk distributions remain unchanged, apart from the absence of foot and tentacle structures, confirming that the foot and tentacle modules are purely morphological features and do not affect axis patterning. **Fig J. Simulations of temporal aspects of head inhibition.** Numerical simulation of the evolution of joined *Hydra* tubes using the one-dimensional model (S2). Parameter values are given in Table A, except for $\tau_{5}=100$, which was increased to slow down the evolution of the source density. The initial data (left) are taken as small perturbations of the homogeneous steady state for all components except the source density. For the source density, initial concentration profiles are extracted from the pattern shown in Fig B and connected to mimic the situation without a head (upper row) and with a head at the right end of the tube (lower row). Blue curves show the evolving concentration profiles, while red curves denote the initial concentration values. **Fig K. Simulations of head formation frequency in repeated ring grafts.** Numerical simulation of the evolution of joined *Hydra* pieces (rings) using the one-dimensional model (S2). Parameter values are taken from Table A, except for $\tau_{5}=5$, which was increased to slow down the evolution of the source density. Initial source-density profiles are extracted from the pattern shown in Fig B and concatenated to mimic a chain of pieces. All other components are initialised as small fluctuations around zero. To simulate the wound response, perturbations of [β] concentrations are added at the junction sites. The spatial domain consists of 25 pieces, each representing approximately one-eighth of the body column. The upper row shows simulations for grafts composed of mid-body pieces, whereas the lower row corresponds to grafts assembled from pieces just below the head. Red curves denote the initial concentration profiles, and blue curves show the final profiles.(PDF)
