# Supplementary material for: Mutual inhibition model of pattern formation: The role of Wnt-Dickkopf interactions in driving Hydra body axis formation
Source: PLoS Comput Biol. 2026 Jul 13;22(7):e1014416. doi: 10.1371/journal.pcbi.1014416 (PMC13384401; doi:10.1371/journal.pcbi.1014416)
Supplement: S1 Appendix — Values of model parameters obtained by fitting the one-dimensional model (S2) to the pattern data obtained from numerical integration of the pseudo-3D model. Table B. Baseline parameter values used for the pseudo-3D simulations. Baseline parameter values used for the pseudo-3D simulations, including the mutual inhibition (MI) model and the auxiliary tentacle and foot modules. Fig A. Intersections of functions f1(w) and f2(w). Intersections of the functions f1(w) and f2(w) for different values of the model parameters ζi, i=1,…,6. The value of ζ1 is indicated in each panel, while all other parameters are fixed to the values given in Table A. Fig B. Comparison of one-dimensional steady-state patterns with pseudo-3D simulation data. Steady-state pattern obtained from the numerical simulation of the one-dimensional model (S2) with the parameter values from Table A, compared with concentration profiles from the pseudo-3D simulations averaged in the direction perpendicular to the body axis. Both concentration profiles are scaled to the interval [0,1] using min–max normalisation. Fig C. Examples of pattern formation for different initial conditions. Three examples of pattern formation in the one-dimensional model. Parameter values used in the simulations are given in Table A. Initial data are taken as small perturbations of the homogeneous steady state. Blue curves show the final concentration profiles, while red curves denote the initial concentration values. Fig D. Semi-analytical bifurcation analysis of the one-dimensional system. Results of the semi-analytical bifurcation analysis of the one-dimensional system. (A) Real parts of the eigenvalues of the linear operator LN(νcr). Real eigenvalues are shown in blue, while the real parts of complex eigenvalues are shown in green. The critical eigenvalue is highlighted in red. (B) Unstable modes as functions of the diffusion coefficient of [W]. (C) Asymptotics of the secondary stationary solution for the first component, evalu [file pcbi.1014416.s001.pdf]

S1 Appendix

Mutual inhibition model of pattern formation: The role of Wnt-Dickkopf interactions in driving *Hydra* body axis formation

Moritz Mercker[1,*], Alexey Kazarnikov[1], Anja Tursch[2], Thomas Richter[3], Suat Özbek[2], Thomas Holstein[2], Anna Marciniak-Czochra[1]

1 Institute for Mathematics and Interdisciplinary Center of Scientific Computing (IWR), Heidelberg University, Heidelberg, Germany

2 Centre for Organismal Studies (COS), Heidelberg University, Heidelberg, Germany

3 Institute of Analysis and Numerics, University Magdeburg, Magdeburg, Germany

*** mmercker_bioscience@gmx.de**

One-dimensional model reduction

Before presenting the mathematical formulation, it is important to note that the model is defined on a functional rather than molecular level. Each variable represents the aggregate activity of several signaling components acting at similar spatial and functional scales, rather than a single gene or protein species. For instance, $[\beta]$ and $[W]$ denote local and diffusible Wnt-related activities, respectively, combining the contributions of multiple Wnt mRNAs and proteins as well as β-catenin and Tcf. The Dickkopf components correspond to the activities of HyDkk1/2/4-A and HyDkk1/2/4-C. Consequently, the model equations describe effective regulatory interactions and are not intended to represent mass-conserving fluxes or stoichiometric relations between individual molecular species.

We consider one-dimensional spatial domain $\Omega = [0, 1]$. The system (1)-(5) (main manuscript) reads

$$\begin{aligned}
\partial_t[\beta] &= \frac{b_1[S]}{(1+k_1[A])(1+k_2[C])(1+k_3[\beta])} - c_1[\beta];\\
\partial_t\,[A] &= a_2\frac{\partial}{\partial x^2}[A] + \frac{b_2}{(1+k_4[\beta])} - c_2[A];\\
\partial_t[W] &= a_3\frac{\partial}{\partial x^2}[W] + b_3[\beta][S] - c_3[W];\\
\partial_t[C] &= a_4\frac{\partial}{\partial x^2}[C] + \frac{b_4[W]}{(1+k_5[\beta])} - c_4[C];\\
\partial_t[S] &= a_5\frac{\partial}{\partial x^2}[S] + b_5[\beta] - c_5[S],
\end{aligned} \tag{S1}$$

supplemented with initial conditions and homogeneous Neumann (zero-flux) boundary conditions for all diffusing components.

To streamline model analysis, we reduce the number of parameters by deriving the system's non-dimensionalised form. To this end, we rescale the variables

$$\alpha_5[\beta] = [\tilde{\beta}],\ k_1[A] = [\tilde{A}],\ \alpha_4 k_2[W] = [\tilde{W}],\ k_2[C] = [\tilde{C}],$$

where $\alpha_i = \dfrac{b_i}{c_i}$, $i = 1, \ldots, 5$, rescale time $\tilde{t} = c_1 t$ and define effective parameters,

$$\zeta_1 = \alpha_2 k_1, \quad \zeta_2 = \frac{\alpha_3\alpha_4 k_2}{\alpha_5}, \quad \zeta_3 = \frac{k_3}{\alpha_5}, \quad \zeta_4 = \frac{k_4}{\alpha_5}, \quad \zeta_5 = \frac{k_5}{\alpha_5}, \quad \zeta_6 = \alpha_1\alpha_5,$$

$$\nu_2 = \frac{a_2}{c_2},\ \nu_3 = \frac{a_3}{c_3},\ \nu_4 = \frac{a_4}{c_4},\ \nu_5 = \frac{a_5}{c_5}.$$

The rescaled system takes the form

$$\boldsymbol{T}\frac{\partial \boldsymbol{w}}{\partial t} = \boldsymbol{D}\frac{\partial^2 \boldsymbol{w}}{\partial x^2} + \boldsymbol{F}(\boldsymbol{w}, \boldsymbol{\zeta}), \tag{S2}$$

where

$$\boldsymbol{w} = \left([\beta](x,t), [A](x,t), [W](x,t), [C](x,t), [S](x,t)\right), \tag{S3}$$

$$\boldsymbol{T} = diag(1, \tau_2, \dots, \tau_5), \tau_i = \frac{c_1}{c_i}, i = 2, \dots, 5, \quad \boldsymbol{D} = diag(0, \nu_2, \dots, \nu_5), \tag{S4}$$

$$\boldsymbol{F}(\boldsymbol{w}, \boldsymbol{\zeta}) = \begin{pmatrix} \dfrac{\zeta_6[S]}{(1+[A])(1+[C])(1+\zeta_3[\beta])} - [\beta] \\ \dfrac{\zeta_1}{(1+\zeta_4[\beta])} - [A] \\ \zeta_2[\beta][S] - [W] \\ \dfrac{[W]}{(1+\zeta_5[\beta])} - [C] \\ [\beta] - [S] \end{pmatrix}, \tag{S5}$$

with $\boldsymbol{\zeta} = (\zeta_1, \dots, \zeta_6)$ being the obtained above non-dimensionalised effective model parameters.

Spatially homogeneous steady states and their stability

Solving the algebraic equations for spatially uniform steady states, we obtain

$$\zeta_6[S] = [\beta](1+[A])(1+[C])(1+\zeta_3[\beta]), \tag{S6}$$

with

$$[A] = \frac{\zeta_1}{1+\zeta_4[\beta]},\ [W] = \zeta_2[\beta]^2,\ [C] = \frac{\zeta_2[\beta]^2}{1+\zeta_5[\beta]},\ [S] = [\beta]. \tag{S7}$$

Substituting (S7) to (S6) and denoting $w := [\beta]$ results in

$$\zeta_6 w = w(1 + \frac{\zeta_1}{1+\zeta_4 w})(1 + \frac{\zeta_2 w^2}{1+\zeta_5 w})(1+\zeta_3 w). \tag{S8}$$

Eq. (S8) has a trivial solution $w = 0$, which corresponds to the semi-trivial steady state $\boldsymbol{w}_0$ with components

$$[\beta] = 0,\ [A] = \zeta_1,\ [W] = 0,\ [C] = 0,\ [S] = 0. \tag{S9}$$

Positive homogeneous steady states satisfies

$$\zeta_6(1+\zeta_4 w)(1+\zeta_5 w) = (1+\zeta_1+\zeta_4 w)(1+\zeta_5 w+\zeta_2 w^2)(1+\zeta_3 w), \tag{S10}$$

and can be found as the intersection points of $f_1(w)$ and $f_2(w)$, where

$$f_1(w) = \zeta_6(1+\zeta_4 w)(1+\zeta_5 w), \tag{S11}$$

and

$$f_2(w) = (1+\zeta_1+\zeta_4 w)(1+\zeta_5 w+\zeta_2 w^2)(1+\zeta_3 w). \tag{S12}$$

Depending on model parameters, there can be a different number of intersections of these functions (Fig A). It holds

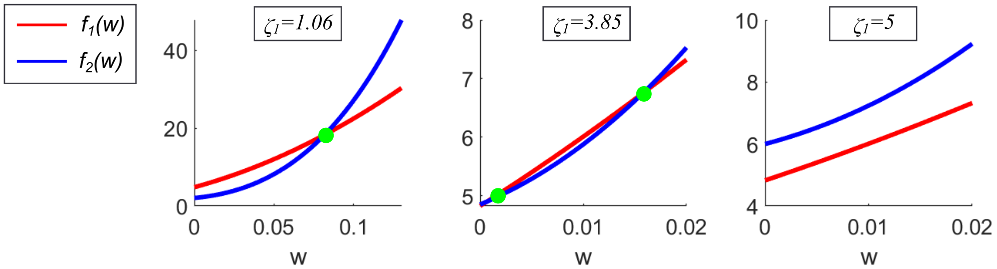

Fig A. Intersections of functions $f_1(w)$ and $f_2(w)$ for different values of model parameters ζ_i, $i = 1, \ldots, 6$. The value of parameter ζ_1 is given in each sub-figure, while all other parameters are fixed to the values given in Table A.

Lemma S1. *The reduced model* (S2) *has no more than three non-trivial spatially homogeneous steady states.*

Proof. $f(w) = f_2(w) - f_1(w) = 0$ is a polynomial with $Deg(f(w)) = 4$. Thus, depending on model parameters, it can have no more than four roots. Furthermore, taking into account that coefficients at the two highest variable exponents are positive and applying Descartes' rule of signs, we conclude that the maximal number of coefficient sign changes and positive real roots is equal to three. □

Linearisation of (S2) at w_0 results in

$$\boldsymbol{T}\frac{\partial \boldsymbol{w}}{\partial t} = \boldsymbol{D}\frac{\partial^2 \boldsymbol{w}}{\partial x^2} + \boldsymbol{N_1}(\boldsymbol{\zeta})\boldsymbol{w}, \tag{S13}$$

where

$$\boldsymbol{N_1}(\boldsymbol{\zeta}) = \frac{\partial \boldsymbol{N_1}(\boldsymbol{w}, \boldsymbol{\zeta})_i}{\partial \boldsymbol{w}_j}|_{\boldsymbol{w}=\boldsymbol{w}_0} = \begin{pmatrix} -1 & 0 & 0 & 0 & \frac{\zeta_6}{1+\zeta_1} \\ -\zeta_1\zeta_4 & -1 & 0 & 0 & 0 \\ 0 & 0 & -1 & 0 & 0 \\ 0 & 0 & 1 & -1 & 0 \\ 1 & 0 & 0 & 0 & -1 \end{pmatrix}. \tag{S14}$$

We define a linear operator

$$L_N := \{\boldsymbol{D}\frac{\partial^2}{\partial x^2} + \boldsymbol{N_1} \text{ with homogeneous Neumann boundary conditions}\} \tag{S15}$$

Applying the spectral decomposition of the Laplace operator with homogeneous Neumann boundary conditions in $L^2(\Omega)$, we can characterise the spectrum of L_N by the spectrum of

$$\tilde{\boldsymbol{N_1}} := -\boldsymbol{D}\lambda_k + \boldsymbol{N_1},$$

where $\lambda_k = (\pi k)^2$, $k = 0, 1, \ldots$, and $\psi_0(x) = 1$, $\psi_k(x) = \sqrt{2}\cos(\pi k x), k = 1, 2, \ldots$, denote the eigenvalues and eigenfunctions of the Laplace operator $-\frac{\partial^2}{\partial x^2}$, defined on the interval $(0, 1)$ with homogeneous Neumann boundary conditions,

$$-\frac{d^2}{dx^2}\psi_k(x) = \lambda_k\psi_k(x), \quad \frac{d}{dx}\psi_k(0) = \frac{d}{dx}\psi_k(1) = 0, \quad k = 0, 1, \ldots. \tag{S16}$$

This reduces the linearised stability problem to the analysis of the characteristic polynomials

$$\varphi_k(\sigma) = det(\boldsymbol{T}^{-1}(\boldsymbol{N_1}(\boldsymbol{\zeta}) - \lambda_k\boldsymbol{D}) - \sigma_k I) = 0, \quad k = 0, 1, \ldots, \tag{S17}$$

which can be rewritten as

$$\varphi_k(\sigma) = -(\sigma+\frac{1+\nu_2\lambda_k}{\tau_2})(\sigma+\frac{1+\nu_3\lambda_k}{\tau_3})(\sigma+\frac{1+\nu_4\lambda_k}{\tau_4})(\sigma^2+(1+\frac{1+\nu_5\lambda_k}{\tau_5})\sigma+\frac{1+\nu_5\lambda_k}{\tau_5}-\frac{\zeta_6}{\tau_5(1+\zeta_1)}). \tag{S18}$$

It yields the stability conditions

$$1+\nu_5\lambda_k - \frac{\zeta_6}{1+\zeta_1} > 0, \quad k = 0, 1, \ldots,$$

which for $k = 0$ read,

$$1 - \frac{\zeta_6}{1+\zeta_1} > 0 \Rightarrow \zeta_6 < 1 + \zeta_1.$$

Consequently, we conclude that the semi-trivial steady state (S9) is always locally asymptotically stable or unstable, i.e. cannot exhibit the Turing instability.

Lemma S2. *If trivial steady state* (S9) *is linearly unstable, then there exists at least one non-trivial spatially homogeneous steady state.*

Proof. The sufficient condition for linear instability reads

$$\zeta_6 > 1 + \zeta_1.$$

However, in this case

$$f_1(0) = \zeta_6, \quad f_2(0) = 1 + \zeta_1,$$

and we have $f_1(0) > f_2(0)$. As $Deg(f_1(w)) = 2$, $Deg(f_2(w)) = 4$, and both polynomials have positive coefficients at leading variable exponents, functions $f_1(w)$ and $f_2(w)$ must have at least one intersection for $w > 0$. □

Lemma S3. *Let $\zeta_4 \leq \zeta_5$, $\zeta_3 < \zeta_5$, and $\zeta_6 > 1 + \zeta_1$. Then, model* (S1) *has exactly one non-trivial spatially homogeneous steady state.*

Proof. Let $w^1 = -\frac{1}{\zeta_4}$ and $w^2 = -\frac{1+\zeta_1}{\zeta_4}$, so that $w^2 < w^1$. Under the assumptions on the limiting constants, we have $f_1(w^1) = 0$ and $f_2(w^1) > 0$, while $f_1(w^2) > 0$ and $f_2(w^2) = 0$. Since both functions are continuous, there must exist at least one point of intersection of $f_1(w)$ and $f_2(w)$ in the interval $[w^2, w^1]$.

Furthermore, since $\deg(f_1(w)) = 2$, $\deg(f_2(w)) = 4$ and both polynomials have positive leading coefficients, additional intersection must occur for $w < w^2$. Additionally, under the assumptions on the model parameters, we have $f_2(0) < f_1(0)$, indicating another point of intersection for negative values of w. Therefore, there can be at most one intersection of $f_1(w)$ and $f_2(w)$ for positive values of w. From Lemma S2, it follows that such an intersection indeed exists. □

Lemma S4. *Let*

$$\begin{aligned} &\zeta_1 + 1 > \zeta_6; \\ &\zeta_4 + (\zeta_3 + \zeta_5)(\zeta_1 + 1) > \zeta_6 (\zeta_4 + \zeta_5); \\ &(\zeta_1 + 1)(\zeta_2 + \zeta_3 \zeta_5) + \zeta_4 (\zeta_3 + \zeta_5) > \zeta_4 \zeta_5 \zeta_6. \end{aligned} \tag{S19}$$

Then, there are no non-trivial steady states of model (S2).

Proof. Let us consider the function used to identify homogeneous steady states: $f(w) = f_2(w) - f_1(w)$. The conditions specified in (S19) ensure that all coefficients of this polynomial are positive. As a consequence, the polynomial $f(w)$ has no positive real roots. Hence, the system admits only the trivial steady state. Furthermore, this steady state is linearly stable, as guaranteed by the first condition in (S19). It follows that all solutions of the model asymptotically approach the trivial steady state. □

Parameter name	Parameter value	Parameter name	Parameter value
ν_1	0	ζ_1	1.06
ν_2	3.81×10^{-5}	ζ_2	540.4
ν_3	4.43×10^{-1}	ζ_3	1.15
ν_4	6.07×10^{-8}	ζ_4	11.59
ν_5	4.78×10^{-4}	ζ_5	11.59
τ_i, $i = 1, \ldots, 5$	1	ζ_6	4.82

Table A. Values of model parameters, obtained by fitting the one-dimensional model (S2) to the 3D pattern data obtained from numerical integration of the 3D model.

Corollary S1. *Let us assume that the following conditions are satisfied:*

$$\begin{aligned} &\zeta_1 + 1 < \zeta_6, \\ &(\zeta_1 + 1)(\zeta_2 + \zeta_3 \zeta_5) + \zeta_4 (\zeta_3 + \zeta_5) > \zeta_4 \zeta_5 \zeta_6. \end{aligned} \tag{S20}$$

Under these conditions, there exists exactly one non-trivial steady state. In particular, the system does not admit bistability.

Proof. The conditions in (S20) ensure that the function $f(w)$ is strictly convex and satisfies $f(0) < 0$. Consequently, $f(w)$ has exactly one positive real root. □

Comparing one-dimensional model with three-dimensional simulations

Before conducting a numerical analysis of pattern formation, it is necessary to determine parameter values for the one-dimensional model (S2) that yield patterns qualitatively similar to those observed in 3D model simulations. The model fitting procedure is as follows. First, the data obtained from the 3D model simulations are pre-processed by averaging the concentration values in the direction perpendicular to the body axis and scaling the resulting one-dimensional profiles to the unit interval using min-max normalisation. This produces data that is suitable for fitting the one-dimensional model; here, our primary interest is in matching the shapes of the gradient profiles rather than the precise concentration values.

We define the residual between the output of (S2) and the pattern data in the least-squares sense,

$$S(\boldsymbol{\nu}, \boldsymbol{\zeta}) = \sum_{i=1}^{M_{\text{dim}}} \left\| \bar{\boldsymbol{w}}_i(\boldsymbol{\nu}, \boldsymbol{\zeta}) - \bar{\boldsymbol{w}}_i^0 \right\|^2, \tag{S21}$$

where $\bar{\boldsymbol{w}}_i^0$ denotes the pre-processed pattern data from the 3D model evaluated at the grid points x_i, for $i = 1, \ldots, M_{\text{dim}}$, and $\bar{\boldsymbol{w}}_i(\boldsymbol{\nu}, \boldsymbol{\zeta})$ is the numerical approximation of the stationary solution of the spatially discretised one-dimensional model with the corresponding parameter values. The initial conditions for the numerical simulation are defined by applying small perturbations to the homogeneous steady state for all model components except for the source density, for which a gradient is prescribed

$$[SD_0](x) = [SD_h] + 4\,\exp(x),$$

where $[SD_h]$ is the corresponding component of the homogeneous steady state. This enforces convergence of the numerical solution to the desired pattern, thereby reducing the variability of the residual function $S(\boldsymbol{\nu}, \boldsymbol{\zeta})$ and simplifying the optimisation process. Subsequently, we minimise the residual function (S21) to obtain optimal values for the diffusion coefficients ν_i and reaction rates ζ_i, for $i = 1, \ldots, 5$, corresponding to a local minimum of the residual. In this step, we set $\tau_i = 1$, for $i = 1, \ldots, 5$, to reduce the dimensionality of the optimisation problem. The estimated model parameters are listed in Table A, and the resulting concentration profiles are illustrated in Fig B.

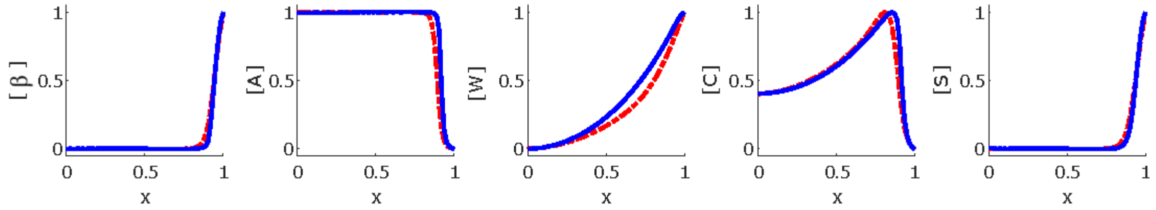

Fig B. Steady-state pattern obtained from the numerical simulation of the one-dimensional model (S2) with the parameter values from Table A (blue) and concentration profiles from the 3D simulations, averaged in the direction perpendicular to the body axis (red). Both concentration profiles are scaled to the interval $[0, 1]$ with the min-max normalisation procedure.

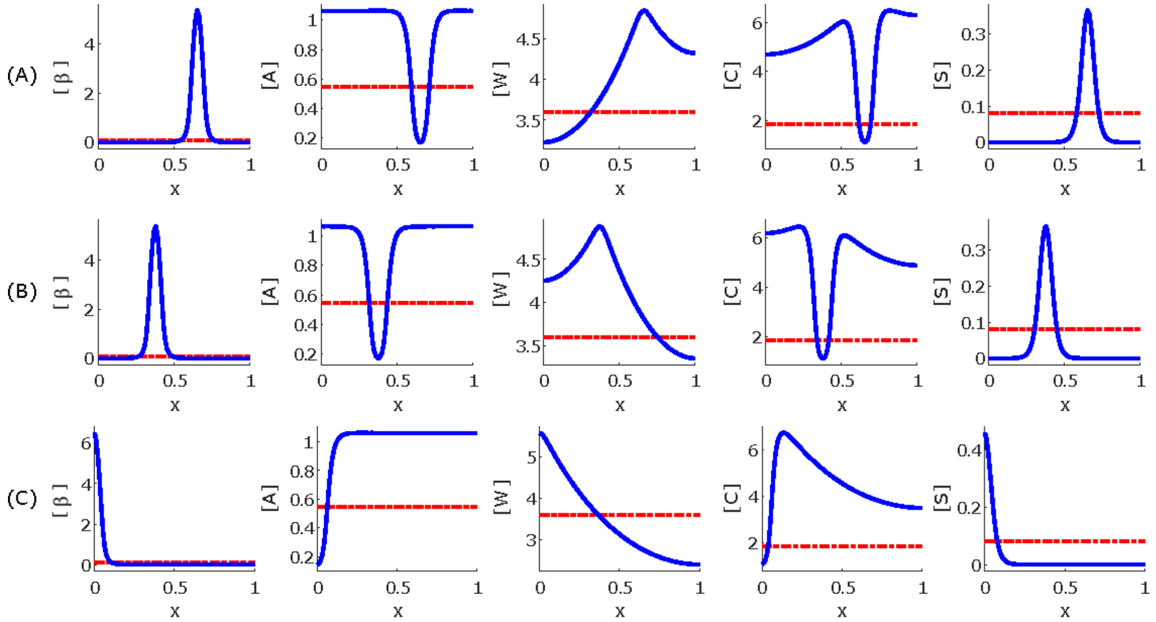

Fig C. Three examples of pattern formation in the one-dimensional model (A-C). Parameter values used in the simulations are given in Table A. Initial data are taken as small perturbations of the homogeneous steady state. Blue colour shows the final concentration profiles, while red colour denotes the initial concentration values.

The obtained model parameters satisfy the conditions of Lemma S3. Consequently, the trivial steady state (S9) is linearly unstable, and there exists a unique non-trivial spatially homogeneous steady state, denoted by $\boldsymbol{w}_1$, which can be computed numerically as

$$\boldsymbol{w}_1 \approx (0.08,\ 0.54,\ 3.60,\ 1.85,\ 0.08).$$

Numerical analysis reveals that $\boldsymbol{w}_1$ exhibits a Turing instability. In all our numerical experiments, regardless of the initial data chosen, the numerical solution consistently converged to spatially inhomogeneous patterns. The resulting concentration profiles are shown in Fig B. However, the shape of the resulting patterns may depend on the initial data, as illustrated in Fig J.

Semi-analytical approach to the analysis of branching patterns

To decipher the nature of the patterns observed in simulations, we analyse the stability of branching stationary solutions near the Turing bifurcation point. As the complexity of the model equations precludes a fully rigorous analytical treatment, we adopt a hybrid approach that combines analytical techniques with numerical methods. In particular, we compute numerical approximations for

quantities that cannot be determined analytically, such as the coordinates of steady states and the eigenvalues of the linearised system.

We apply the classical Lyapunov-Schmidt method [1, 2] to investigate the branching behaviour of stationary solutions of the one-dimensional model (S2). To streamline the analysis, we set $\boldsymbol{T} = \boldsymbol{I}$. We assume that all parameters are fixed except for the largest diffusion coefficient, $\nu_3 := \nu$, which we treat as a bifurcation parameter. Expanding the nonlinearity $\boldsymbol{N}(\boldsymbol{w}, \boldsymbol{\zeta})$ in a Taylor series about a spatially homogeneous steady state $\boldsymbol{w}_1$, we obtain

$$\frac{\partial \boldsymbol{u}}{\partial t} = \boldsymbol{D}(\nu)\frac{\partial^2 \boldsymbol{u}}{\partial x^2} + \boldsymbol{N}_1(\boldsymbol{u}) + \boldsymbol{N}_2(\boldsymbol{u},\boldsymbol{u}) + \boldsymbol{N}_3(\boldsymbol{u},\boldsymbol{u},\boldsymbol{u}) + \dots, \quad \boldsymbol{w} = \boldsymbol{w}_1 + \boldsymbol{u}, \tag{S22}$$

where the linear operator $\boldsymbol{N}_1$ is defined by the Jacobian matrix (S14), and the higher-order terms $\boldsymbol{N}_2$ and $\boldsymbol{N}_3$ are given by

$$(\boldsymbol{N}_2)_j(\boldsymbol{u},\boldsymbol{u}) = \frac{1}{2}\sum_{i_1,i_2=1}^{5} u_{i_1}u_{i_2}\,\frac{\partial^2 \boldsymbol{N}_j}{\partial u_{i_1}\partial u_{i_2}}\bigg|_{\boldsymbol{u}=0}, \quad j = 1,\dots,5,$$

$$(\boldsymbol{N}_3)_j(\boldsymbol{u},\boldsymbol{u},\boldsymbol{u}) = \frac{1}{6}\sum_{i_1,i_2,i_3=1}^{5} u_{i_1}u_{i_2}u_{i_3}\,\frac{\partial^3 \boldsymbol{N}_j}{\partial u_{i_1}\partial u_{i_2}\partial u_{i_3}}\bigg|_{\boldsymbol{u}=0}, \quad j = 1,\dots,5.$$

Next, we consider a linear spectral problem for the operator $L_N(\nu)$, defined in (S15), on $L^2(\Omega)$,

$$L_N(\nu)\boldsymbol{\phi} = \sigma\boldsymbol{\phi},\ \boldsymbol{\phi} \neq 0, \tag{S23}$$

and seek an eigenfunction $\boldsymbol{\phi}$ in the form of a series expansion with vector coefficients,

$$\boldsymbol{\phi} = \sum_{k=0}^{+\infty} \boldsymbol{C}_k \psi_k(x), \quad \boldsymbol{C}_k \in \mathbb{R}^5. \tag{S24}$$

By substituting (S24) into (S23) and matching coefficients of identical eigenfunctions, we derive a sequence of linear systems,

$$\boldsymbol{N}_1 - \boldsymbol{D}(\nu)\lambda_k \boldsymbol{I} = 0, \quad k = 0, 1, \dots, \tag{S25}$$

from which the eigenvalues $\sigma_j^k(\nu)$, for $j = 1,\dots,5$, of the operator $L_N(\nu)$ can be determined.

The critical value ν_{cr} of the control parameter ν is defined as the value at which the spectrum of the linear operator $L_N(\nu_{\mathrm{cr}})$ lies entirely within the closed left half-plane of the complex plane, with at least one eigenvalue σ located on the imaginary axis and satisfying

$$\frac{d\,\mathrm{Re}(\sigma)}{d\nu}\bigg|_{\nu=\nu_{\mathrm{cr}}} \neq 0.$$

We recall that the spatially homogeneous steady state $\boldsymbol{w}_1$ is said to be *Turing unstable* if the following conditions are satisfied:

1. All eigenvalues σ_j^0, for $j = 1,\dots,5$, of the linearised system *without* diffusion lie strictly in the left half-plane of the complex plane.
2. There exists at least one eigenvalue $\sigma_{j_0}^{k_0}$, with $k_0 > 0$, of the linearised system *with* diffusion that lies in the right half-plane of the complex plane.

If the only eigenvalue on the imaginary axis at $\nu = \nu_{\rm cr}$ is $\sigma = 0$, the system undergoes a *monotonic* (or *stationary*) instability. In contrast, if a pair of purely imaginary eigenvalues $\sigma = \pm i\omega_0$ exists at $\nu = \nu_{\rm cr}$, the system exhibits an *oscillatory* instability, commonly referred to as a *Hopf bifurcation*.

Assuming that $\nu_{\rm cr}$ is the critical value of the control parameter ν such that there exists a single simple eigenvalue $\sigma_{j_0}^{k_0}(\nu_{\rm cr}) = 0$, while all other eigenvalues lie strictly in the left half-plane of the complex plane, we construct an asymptotic approximation of the branching stationary solutions using the Lyapunov–Schmidt reduction method. As a first step, we seek the eigenfunctions of the linearised spectral problem and its adjoint:

$$L_N(\nu_{\rm cr})\boldsymbol{\varphi} = 0, \quad L_N^*(\nu_{\rm cr})\boldsymbol{\Phi} = 0, \quad \langle \boldsymbol{\varphi}, \boldsymbol{\Phi} \rangle = 1,$$

where $\boldsymbol{\varphi}$ and $\boldsymbol{\Phi}$ are the eigenfunctions of the operator $L_N(\nu_{\rm cr})$ and its adjoint $L_N^*(\nu_{\rm cr})$, respectively. The inner product is normalised such that $\langle \boldsymbol{\varphi}, \boldsymbol{\Phi} \rangle = 1$. Due to the simplicity of $\sigma_{j_0}^{k_0}(\nu_{\rm cr})$, these functions can be expressed as

$$\boldsymbol{\varphi} = \boldsymbol{b}\,\psi_{k_0}(x), \quad \boldsymbol{\Phi} = \boldsymbol{c}\,\psi_{k_0}(x). \tag{S26}$$

Next, we define in (S22) the small parameter $\varepsilon^2 = \nu - \nu_{\rm cr}$, set $\boldsymbol{u}_t = 0$, and obtain the stationary equation,

$$0 = L_N(\nu_{\rm cr})\boldsymbol{u} + \varepsilon^2 \boldsymbol{B}\boldsymbol{u} + \boldsymbol{N}_2(\boldsymbol{u}, \boldsymbol{u}) + \boldsymbol{N}_3(\boldsymbol{u}, \boldsymbol{u}, \boldsymbol{u}) + \ldots, \tag{S27}$$

where $\boldsymbol{B}\boldsymbol{u} = \boldsymbol{D}_1 \dfrac{d^2\boldsymbol{u}}{dx^2}$ and $\boldsymbol{D}_1 = \mathrm{diag}(0, 0, 1, 0, 0)$.

Branching stationary solutions for $\varepsilon > 0$ are constructed in the form of a power series:

$$\boldsymbol{u} = \sum_{k=1}^{\infty} \varepsilon^k \boldsymbol{u}_k. \tag{S28}$$

By substituting (S28) into (S27) and equating the coefficients of like powers of ε, we obtain the following chain of equations:

$$\varepsilon^1 : \quad -L_N(\nu_{\rm cr})\boldsymbol{u}_1 = 0; \tag{S29}$$

$$\varepsilon^2 : \quad -L_N(\nu_{\rm cr})\boldsymbol{u}_2 = \boldsymbol{N}_2(\boldsymbol{u}_1, \boldsymbol{u}_1) \equiv \boldsymbol{f}_2; \tag{S30}$$

$$\varepsilon^3 : \quad -L_N(\nu_{\rm cr})\boldsymbol{u}_3 = \boldsymbol{B}\boldsymbol{u}_1 + 2\boldsymbol{N}_2(\boldsymbol{u}_1, \boldsymbol{u}_2) + \boldsymbol{N}_3(\boldsymbol{u}_1, \boldsymbol{u}_1, \boldsymbol{u}_1) \equiv \boldsymbol{f}_3. \tag{S31}$$

The solution to equation (S29) is given by the expression:

$$\boldsymbol{u}_1 = \zeta_1 \boldsymbol{\varphi},$$

where the constant ζ_1 will be determined at a later stage of the method. The solvability condition for the inhomogeneous equation (S30) is

$$(\boldsymbol{f}_2, \boldsymbol{\Phi}) = \int_0^1 \left(f_2^1 \Phi_1 + \ldots + f_2^5 \Phi_5 \right) dx = 0,$$

which is automatically satisfied due to the structure of $\boldsymbol{f}_2$. The solution of equation (S30) can then be written as

$$\boldsymbol{u}_2 = \zeta_2 \boldsymbol{\varphi} + \boldsymbol{u}_{20}, \quad \boldsymbol{u}_{20} = \zeta_1^2 \left(\boldsymbol{v}_2^0 + \boldsymbol{v}_2^{2k_0} \psi_{2k_0}(x) \right),$$

where the vectors $\boldsymbol{v}_2^0$ and $\boldsymbol{v}_2^{2k_0}$ are determined as solutions of the following linear systems:

$$N_1 \boldsymbol{v}_2^0 = -\frac{1}{2} N_2(\boldsymbol{b}, \boldsymbol{b}), \quad \left(N_1 - \lambda_{2k_0} \boldsymbol{D}(\nu_{\rm cr})\right) \boldsymbol{v}_2^{2k_0} = -\frac{1}{2} N_2(\boldsymbol{b}, \boldsymbol{b}).$$

The solvability condition for equation (S31) takes the form:

$$(\boldsymbol{f}_3, \boldsymbol{\Phi}) = \zeta_1(B\boldsymbol{\varphi}, \boldsymbol{\Phi}) + 2\zeta_1\zeta_2(N_2(\boldsymbol{\varphi}, \boldsymbol{\varphi}), \boldsymbol{\Phi}) + 2\zeta_1^3 \left(N_2\left(\boldsymbol{\varphi}, \boldsymbol{v}_2^0 + \boldsymbol{v}_2^{2k_0}\psi_{2k_0}(x)\right), \boldsymbol{\Phi}\right)$$
$$+ \zeta_1^3(N_3(\boldsymbol{\varphi}, \boldsymbol{\varphi}, \boldsymbol{\varphi}), \boldsymbol{\Phi}) = 0.$$

From this, we derive the expression for ζ_1^2:

$$\zeta_1^2 = -\frac{(B\boldsymbol{\varphi}, \boldsymbol{\Phi})}{(N_3(\boldsymbol{\varphi}, \boldsymbol{\varphi}, \boldsymbol{\varphi}), \boldsymbol{\Phi}) + 2(N_2(\boldsymbol{\varphi}, \boldsymbol{v}_2^0 + \boldsymbol{v}_2^{2k_0}\psi_{2k_0}(x)), \boldsymbol{\Phi})}, \tag{S32}$$

where

$$(N_3(\boldsymbol{\varphi}, \boldsymbol{\varphi}, \boldsymbol{\varphi}), \boldsymbol{\Phi}) = \frac{3}{8}(N_3(\boldsymbol{b}, \boldsymbol{b}, \boldsymbol{b}), \boldsymbol{c}),$$
$$(N_2(\boldsymbol{\varphi}, \boldsymbol{v}_2^0 + \boldsymbol{v}_2^{2k_0}\psi_{2k_0}(x)), \boldsymbol{\Phi}) = \frac{1}{2}(N_2(\boldsymbol{b}, \boldsymbol{v}_2^0), \boldsymbol{c}) + \frac{1}{4}(N_2(\boldsymbol{b}, \boldsymbol{v}_2^{2k_0}), \boldsymbol{c}),$$
$$(B\boldsymbol{\varphi}, \boldsymbol{\Phi}) = \frac{1}{2}(-\lambda_{k_0} D_1 \boldsymbol{b}, \boldsymbol{c}).$$

Here, the sign of ζ_1^2 determines the linear stability of the branching solutions. If ζ_1^2 is positive, then the pair of branching stationary solutions

$$\boldsymbol{w}_{1,2} = \boldsymbol{w}_1 \pm \varepsilon\zeta_1\boldsymbol{\varphi} + O(\varepsilon^2)$$

is stable for small $\varepsilon = \sqrt{\nu - \nu_{\mathrm{cr}}}$.

To apply the method, we fix the model parameters to the values given in Table A. The steady state $\boldsymbol{w}_1$ is found using Newton's method:

$$\boldsymbol{w}_1 \approx (0.0816, 0.5458, 3.6049, 1.8513, 0.0816).$$

The critical value of the control parameter ν_{cr} is estimated by considering the eigenvalue problem (S23) and truncating the series expansion (S24) with a sufficiently large cut-off value $N = 100$:

$$\boldsymbol{u} \approx \sum_{k=0}^{N} \boldsymbol{C}_k \phi_k(x). \tag{S33}$$

Substituting (S33) into (S23), we obtain a finite number of linear systems:

$$\boldsymbol{N}_1 - \boldsymbol{D}(\nu)\lambda_k \boldsymbol{I} = 0, \quad k = 0, 1, 2, \ldots, N,$$

from which we numerically determine the eigenvalues $\sigma_j^k(\nu)$. We introduce the residual function

$$f(\nu) = \max\left|\sigma_j^k(\nu)\right|, \quad k = 0, \ldots, N,\ j = 1, \ldots, 5,$$

and minimize it with respect to ν using standard nonlinear optimization methods. The result is

$$\nu_{\mathrm{cr}} \approx 0.0094.$$

The first eigenvalues of the operator $L_N(\nu_{\mathrm{cr}})$ are shown in Fig D, part A. All plotted values are negative, except for one eigenvalue $\sigma_j^{k_0} \approx 0$, where $k_0 = 8$. Next, we estimate the vectors $\boldsymbol{b}$ and $\boldsymbol{c}$ in the expressions for eigenfunctions (S26),

$$b \approx (-0.2280, 0.7238, -2.5573, 1.2007, -0.1751), \quad c \approx (-2.4022, 0.1239, 0.0050, 0.0688, -1.6726).$$

Next, we evaluate the expression (S32) and obtain $\zeta_1^2 \approx 44.6539 > 0$, meaning that branching stationary solutions are stable (see Fig D, part B). This corresponds to the scenario of Turing pattern formation.

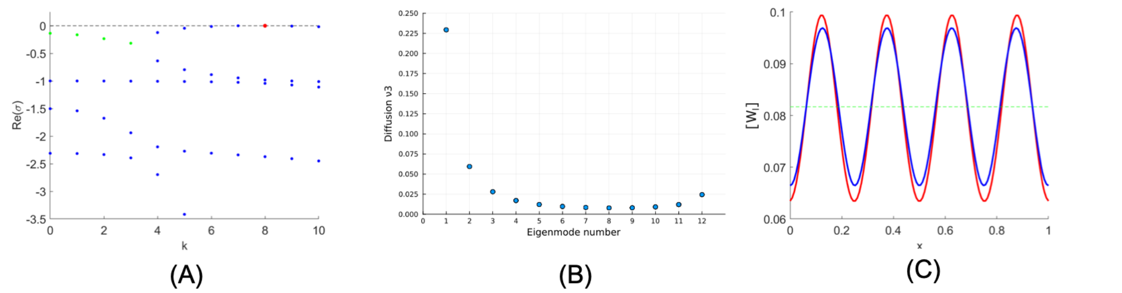

Fig D. The results of a semi-analytical bifurcation analysis of the one-dimensional system. (A): The real parts of the eigenvalues of the linear operator $L_N(\nu_{\rm cr})$. Real eigenvalues are shown in blue, while the real parts of complex eigenvalues are depicted in green. The critical eigenvalue is highlighted in red. (B) Unstable modes in dependence of the diffusion coefficient of $[W]$. (C): The asymptotics of the secondary stationary solution (first component), evaluated at $\varepsilon = 0.01$ (blue), compared to the numerical solution of the system computed with the same parameter values (red). The green dashed line represents the respective component of the spatially homogeneous steady state $\boldsymbol{w}_1$. The initial conditions for the simulation are small random perturbations of the stationary state.

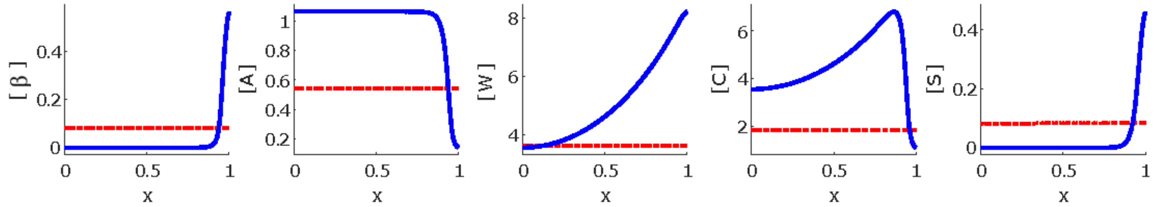

Fig E. Example pattern, obtained by numerically integrating system (S34)-(S38). Initial data are taken as small perturbations of the homogeneous steady state $\boldsymbol{w_1}$. The blue colour shows the final concentration profiles, while the red colour denotes the initial concentration values.

Model variations

To examine the robustness of the one-dimensional model (S1) to changes in reaction terms, we consider two variations of the model. The first version is obtained by multiplying the reaction terms in (S1) by the respective degradation terms, giving the following system of equations,

$$\partial_t[\beta] = \quad b_1[S] - c_1[\beta](1 + k_1[A])(1 + k_2[C])(1 + k_3[\beta]) \tag{S34}$$

$$\partial_t\,[A] = \quad a_2 \frac{\partial}{\partial x^2}[A] + b_2 - c_2[A](1 + k_4[\beta]) \tag{S35}$$

$$\partial_t[W] = \quad a_3 \frac{\partial}{\partial x^2}[W] + b_3[\beta][S] - c_3[W] \tag{S36}$$

$$\partial_t[C] = \quad a_4 \frac{\partial}{\partial x^2}[C] + b_4[W] - c_4[C](1 + k_5[\beta]) \tag{S37}$$

$$\partial_t[S] = \quad a_5 \frac{\partial}{\partial x^2}[S] + b_5[\beta] - c_5[S]. \tag{S38}$$

It can be interpreted as accounting for regulatory feedback in the decay terms.

System (S34)-(S38) has obviously the same spatially homogeneous steady states as the original system (S1). Next, we simulate this system for the parameter values shown in Table A. An example pattern is shown in Fig E.

Another modification was obtained by replacing some reaction terms in system (S1) with higher

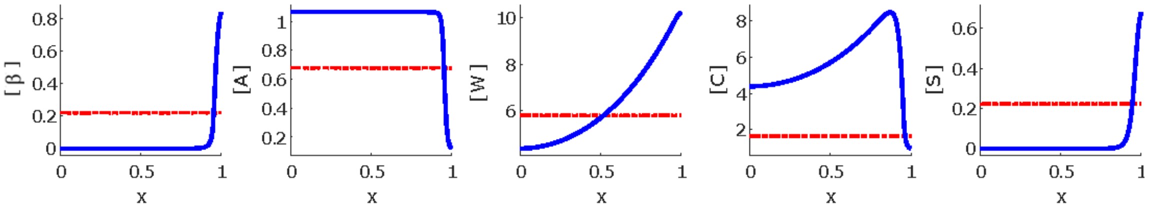

Fig F. Example pattern, obtained by numerically integrating system (S39)-(S43). Initial data are taken as small perturbations of the homogeneous steady state $\boldsymbol{w_1}$. The blue colour shows the final concentration profiles, while the red colour denotes the initial concentration values.

power terms. We arrive at the following system of equations

$$\partial_t[\beta] = \frac{b_1[S]}{(1+k_1[A]^2)(1+k_2[C])(1+k_3[\beta])} - c_1[\beta] \tag{S39}$$

$$\partial_t\,[A] = a_2\frac{\partial}{\partial x^2}[A] + \frac{b_2}{(1+k_4[\beta]^2)} - c_2[A] \tag{S40}$$

$$\partial_t[W] = a_3\frac{\partial}{\partial x^2}[W] + b_3[\beta][S]^2 - c_3[W] \tag{S41}$$

$$\partial_t[C] = a_4\frac{\partial}{\partial x^2}[C] + \frac{b_4[W]}{(1+k_5[\beta])} - c_4[C] \tag{S42}$$

$$\partial_t[S] = a_5\frac{\partial}{\partial x^2}[S] + b_5[\beta] - c_5[S]. \tag{S43}$$

We simulate the model (S39)-(S43) using parameters from Table A. The resulting pattern is shown in Fig F.

Mathematical framework for modelling pseudo-3D geometry

The computational work presented in this paper is based on a mechano-chemical modelling framework [3–5], which couples the mutual inhibition model (MI) of Wnt-Dkk interactions, described by the reaction-diffusion equations in Eq. (1)-(5) (main manuscript), to a continuous tissue evolution model.

The tissue, forming a hollow ellipsoid in the shape of a *Hydra* cell bilayer, is approximated at each time t by a closed 2D surface $\Gamma(t)$ embedded in 3D space. Detailed modelling of the cell layers is neglected. The evolution of $\Gamma(t)$ is represented by a diffeomorphic, time-dependent map $\vec{X}$, parameterised over the unit sphere $S^2 \subset \mathbb{R}^3$, so that $\Gamma(t)$ is the image of $\vec{X}(\cdot, t)$, with $\vec{X}(\vec{s}, t) : S^2 \times [0, T] \to \mathbb{R}^3$ for $T \in \mathbb{R}_{>0}$. The chemical processes on the evolving tissue surface are modelled by identifying material points $\vec{X}(\vec{s}, t)$ on $\Gamma(t)$ with $\vec{s} \in S^2$. The smooth and bijective nature of $\vec{X}$ ensures this correspondence. Functions $\phi_a : S^2 \times [0, T] \to \mathbb{R}_{\geq 0}$, given by $\phi_a(\vec{s}, t) = \Phi_a(\vec{X}(\vec{s}, t))$, describe the local concentrations of gene products, where Φ_a represents the concentrations $([\beta], [A], [W], [C], [S], ...)$ in the MI model.

Assuming purely elastic tissue, the elastic deformations are modelled using Helfrich's free energy [6], defined as:

$$\mathcal{F}_{bend} = \int_\Gamma \kappa\big(H - H_0([\beta], [T_a], [F_a])\big)^2\, d\vec{S},$$

where H is the mean curvature, κ is the bending rigidity, and H_0 represents the spontaneous curvature [3,4]. H_0 is assumed to depend on local morphogen concentrations, and we take $H_0([\beta], [T_a], [F_a]) = 1.5[\beta] + 3.0[F_a] + 10.0[T_a]$, based on local tissue evaginations observed during head, foot, and tentacle

formation (cf., below) [7–9]. The evolution of the tissue surface is computed from the L^2-gradient flow of the total energy, including a local Lagrange multiplier. Minimization of the free energy results in a 4th order PDE model for the deforming tissue surface. This model is coupled to the reaction-diffusion system in Eq. (1)-(5) (main manuscript), with the diffusion process represented by the surface Laplace-Beltrami operator Δ^Γ. For further details, see Ref. [3].

Extended model for tentacle formation

To complement the qualitative description in the main text, we provide here the explicit mathematical formulation of the tentacle subsystem. The tentacle module is phenomenological and represented by an activator–inhibitor system receiving positive input from the source density ($[S]$) and negative input from local Wnt activity ($[\beta]$). This structure captures the experimentally observed formation of tentacle primordia in regions of intermediate head-forming competence, where $[S]$ is high but β-catenin/Wnt locally suppresses/shifts the tentcale system [10–13]. Including the tentacle subsystem does not affect Wnt–Dkk pattern formation but provides additional data for model verification, such as the response to ALP treatment.

$$d_t[T_a] = a_6\Delta^\Gamma[T_a] + \frac{b_6[S](g+[T_a]^2)}{[T_i](e+d[T_a]^2)(e+h[\beta])} - c_6[T_a], \tag{S44}$$

$$d_t[T_i] = a_7\Delta^\Gamma[T_i] + \frac{b_7[S](g+[T_a]^2)}{(e+d[T_a]^2)(e+h[\beta])} - c_7[T_i]. \tag{S45}$$

The activator ($[T_a]$) promotes tentacle initiation, whereas the inhibitor ($[T_i]$) limits its spread, thereby generating a periodic array of tentacle primordia. The system thus provides a minimal description of the tentacle patterning process while remaining functionally decoupled from the Wnt–Dkk axis formation module.

Extended model for foot formation

Analogously, the model was further extended to include a subsystem describing foot formation, formulated as a separate activator–inhibitor pair that interacts with the source density ($[S]$) but not with the Wnt–Dkk core system. The foot subsystem represents the basal organiser region and is used to reproduce realistic *Hydra* morphology and regeneration behaviour. The activator is enhanced under conditions of low $[S]$, consistent with the basal identity being promoted in regions distant from the head organiser [13–15].

$$d_t[F_a] = a_8\Delta^\Gamma[F_a] + \frac{b_8(h+[F_a]^2)}{[F_i][S]} - c_8[F_a], \tag{S46}$$

$$d_t[F_i] = a_9\Delta^\Gamma[F_i] + \frac{b_9(i+[F_a]^2)}{[S]} - c_9[F_i]. \tag{S47}$$

The foot subsystem is purely phenomenological and does not feed back into the body-axis patterning. It stabilises the aboral pole and contributes to the overall morphological realism of the simulated *Hydra* shape. All parameter values and simulation settings are listed below.

Numerical implementation pseudo 3D-model

The mathematical model was simulated using the finite element library Gascoigne [16], based on approximation of the fourth order PDEs in a mixed formulation. We applied linear finite elements for spatial discretisation, and a semi-implicit Euler scheme for time discretisation. For further details of the computation scheme, see [3, 4].

Numerical vs. real time and space units

Above mentioned numerical time and space scales can be roughly related to real scales in the following way: The average size of an adult *Hydra* polyp is approximately $X_{real} = 20\,mm$, which corresponds to the length of $X_{num} = 6$ numerical units, leading to the relation $X_{real} \approx \frac{10}{3} X_{num}\,mm$. With respect to time, only a coarse estimation is possible. During simulations, head regeneration is finished for $T_{num} > 0.002$. In experiments, head regeneration requires approx. 48 hours (e.g., [12,17]), leading to $T_{real} \approx T_{num}/(4.2 \times 10^{-5})\,hrs$.

Parameters and initial conditions

The mathematical model was simulated using the finite element library Gascoigne [16], which approximates the fourth-order PDEs in a mixed formulation. Linear finite elements were used for spatial discretisation, while a semi-implicit Euler scheme was employed for time discretisation. For further details on the computation scheme, see [3, 4].

For simulations of the unperturbed system, all baseline parameter values of the pseudo-3D MI model and the auxiliary tentacle/foot modules are summarised in Table B. Unless explicitly stated otherwise (e.g., HyDkk removal or knockdown; see below), simulations are based on this single baseline parameter set. The tentacle/foot parameters are adapted from [13, 14, 18].

**Subsystem**	**Parameter(s)**	**Baseline value(s)**
MI model	$b_1,\ c_1$	$b_1 = 15 \times 10^{-3}, \quad c_1 = 3 \times 10^{-3}$
MI model	a_1	$a_1 = 18 \times 10^{-5}$ (small diffusion of first variable; numerical stabilisation)
MI model	$a_2,\ b_2,\ c_2$	$a_2 = 18 \times 10^{-7}, \quad b_2 = 3 \times 10^{-3}, \quad c_2 = 4 \times 10^{-3}$
MI model	$a_3,\ b_3,\ c_3$	$a_3 = 22 \times 10^{-3}, \quad b_3 = 3 \times 10^{-3}, \quad c_3 = 4 \times 10^{-3}$
MI model	$a_4,\ b_4,\ c_4$	$a_4 = 1 \times 10^{-7}, \quad b_4 = 1 \times 10^{-2}, \quad c_4 = 1 \times 10^{-2}$
MI model	$a_5,\ b_5,\ c_5$	$a_5 = 11 \times 10^{-6}, \quad b_5 = 3 \times 10^{-4}, \quad c_5 = 3 \times 10^{-4}$
MI model	$d,\ e,\ f$	$d = 0.1, \quad e = 1.0, \quad f = 0.3$
tentacle/foot	$a_6,\ b_6,\ c_6$	$a_6 = 7.5 \times 10^{-5}, \quad b_6 = 2.0 \times 10^{-2}, \quad c_6 = 2.0 \times 10^{-2}$
tentacle/foot	$a_7,\ b_7,\ c_7$	$a_7 = 3.0 \times 10^{-3}, \quad b_7 = 3.0 \times 10^{-2}, \quad c_7 = 3.0 \times 10^{-2}$
tentacle/foot	$a_8,\ b_8,\ c_8$	$a_8 = 7.2 \times 10^{-4}, \quad b_8 = 2.0 \times 10^{-3}, \quad c_8 = 3.0 \times 10^{-3}$
tentacle/foot	$a_9,\ b_9,\ c_9$	$a_9 = 4.4 \times 10^{-2}, \quad b_9 = 2.0 \times 10^{-3}, \quad c_9 = 3.0 \times 10^{-3}$
tentacle/foot	$g,\ h,\ i$	$g = 0.005, \quad h = 0.01, \quad i = 0.0001$

Table B. Baseline parameter values used for the pseudo-3D simulations (MI model and auxiliary tentacle/foot modules).

The numerical values listed in Table B represent effective, coarse-grained parameters of the model and are not intended to correspond to individually measured biochemical rate constants. Their magnitudes are chosen such that (i) the diffusion hierarchy realises local activation and effective long-range inhibition (with a_3 being the dominant diffusion coefficient), and (ii) a clear separation of time scales is achieved, in particular with the source density $[S]$ evolving more slowly than the Wnt–Dkk components (cf. $c_5 \ll c_1,...,c_4$). Importantly, diffusion terms should be interpreted functionally as effective propagation of Wnt-related activity; depending on the biological context, this may reflect extracellular dispersal of ligands, synergistic spread of multiple Wnts, active transport, or non-chemical propagation mechanisms such as biomechanical coupling or bioelectrical signalling [19–25].

Robustness analyses reported within S1 Appendix demonstrate that the qualitative pattern-forming behaviour persists under broad parameter variations over several orders of magnitude. Thus, the relevant structural properties of the model depend on parameter hierarchies and interaction topology rather than on fine-tuned numerical calibration of individual rates.

For the simulations, the geometry of the *Hydra* tissue was approximated by parametrising initial conditions for X_1, X_2, and X_3 over a closed 2D unit sphere S^2 embedded in 3D space, with the

initial condition $X_1(t=0) \equiv X_2(t=0) \equiv 0$ and $X_3(t=0) = 4 \cdot s_3$, leading to a stretch in the s_3 direction (where s_1, s_2, s_3 are Eulerian coordinates of the S^2 surface).

For the variables representing (partially groups of) signalling factors, we used stochastic initial distributions based on the standard random generator provided by C++. Only the initial concentration of [S] was modelled using a gradient defined by $[S](t=0) = 4.0 \cdot (\exp(s_3)/\exp(1))$. For simulations of grafting experiments, $[S](t=0) = 2.0 \cdot (\exp(s_3)/\exp(1))$ was used, representing the oral region with maximal [S] values (the head) being removed. Here, in addition, initial spots of increased [S] values mimicking grafted pieces (including a corresponding wound response), since high values of [S] on the one hand represent tissue from the oral region of the animal, and on the other hand they immediately activate the other head-related molecules representing a wound response. Here, in particular, they were introduced using circles of radius $r = 0.33$ mm, where $[S] = 4.0$. For simulations of ALP treatment (cf., main manuscript), the initial conditions for the source density were modified to $[S](t=0) = 2.0 + 4.0 \cdot (\exp(s_3)/\exp(1))$.

To simulate the complete removal of *HyDkk1/2/4-A* or *HyDkk1/2/4-C* expression at a specific time point (after the head pattern had been established), the parameters b_2 and b_4 were set to zero. HyDkk1/2/4-A knockdown was modeled by increasing c_2 from 4×10^{-3} to 6×10^{-3}.

For *Hydra* aggregates, the initial geometry was a sphere, and all molecules, including [S], were given random initial conditions.

Further supporting simulations of the pseudo 3D model

This section contains various supporting simulations of the pseudo-3D model (and derived 1D simulation profiles) amongst others in comparison to further experimental data.

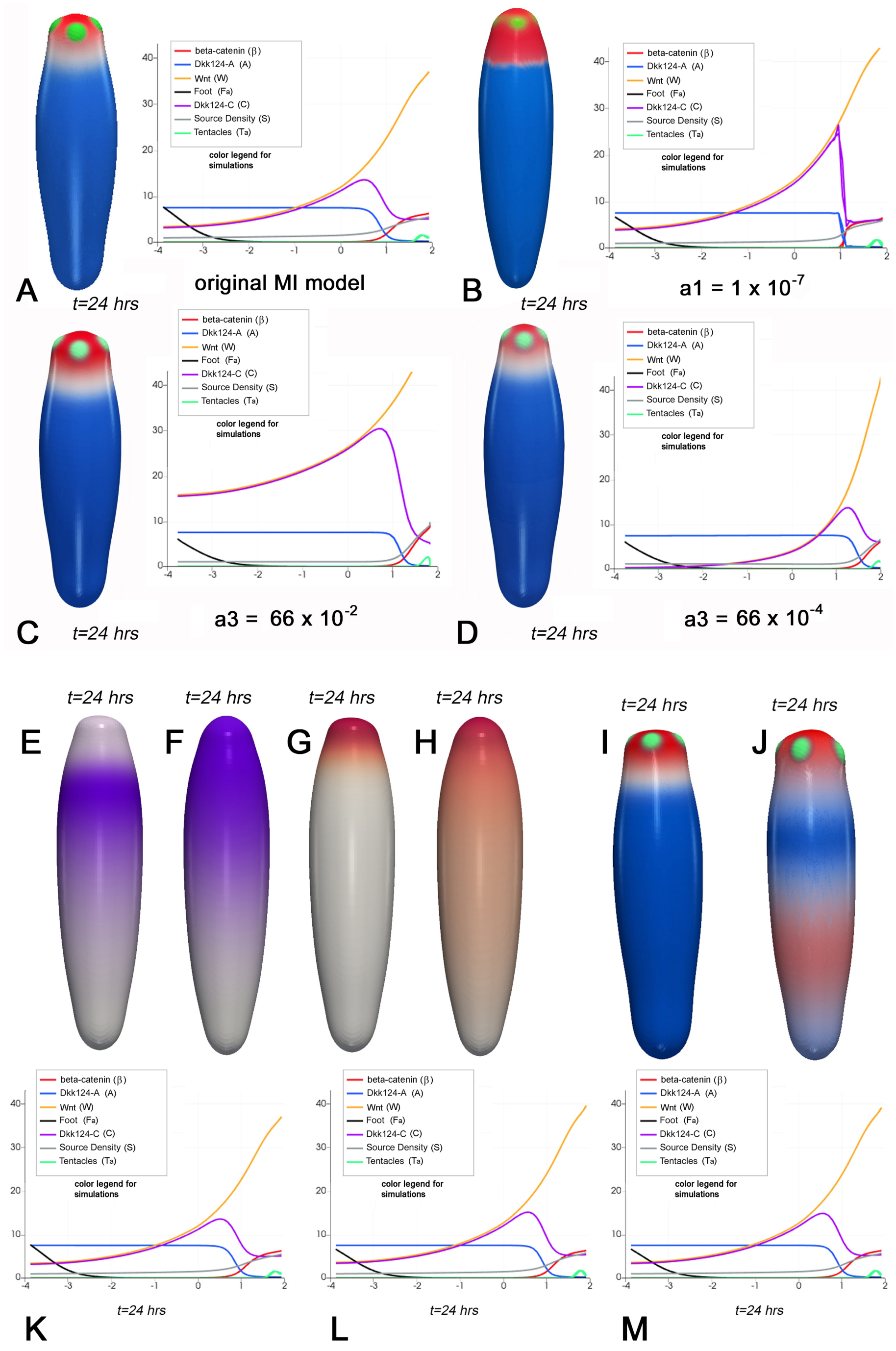

Fig G. (A-D): 3D stable patterns and extracted 1D concentration profiles for models with different diffusion rates of the $[\beta]$ complex (B) or of $[W]$ (C–D) versus the results for the unperturbed system (A). (E-H): Simulated (rescaled) distribution of Dkk1/2/4-C (E–F) and $[\beta]$ (G–H) expression for the undisturbed system (E,G) vs. a system in which no $[\beta]$-based inhibition of Dkk1/2/4-C has been considered (F,H). In particular, in the latter, expression patterns resemble the classical activator-inhibitor model. (I-J): Relative distribution of simulated Dkk1/2/4-A (blue), $[\beta]$ (red), and tentacle (green) expression for the undisturbed system (I) vs. a system without constant *Dkk1/2/4-A*-expression but an [S]-induced expression instead (J). (K-M): Concentration profiles of the unperturbed system (K) versus the system with equal Dkk1/2/4-A and Dkk1/2/4-C diffusion rates (L–M). (L): $a_2 = a_4 = 1 \times 10^{-6}$: (M): $a_2 = a_4 = 1 \times 10^{-7}$.

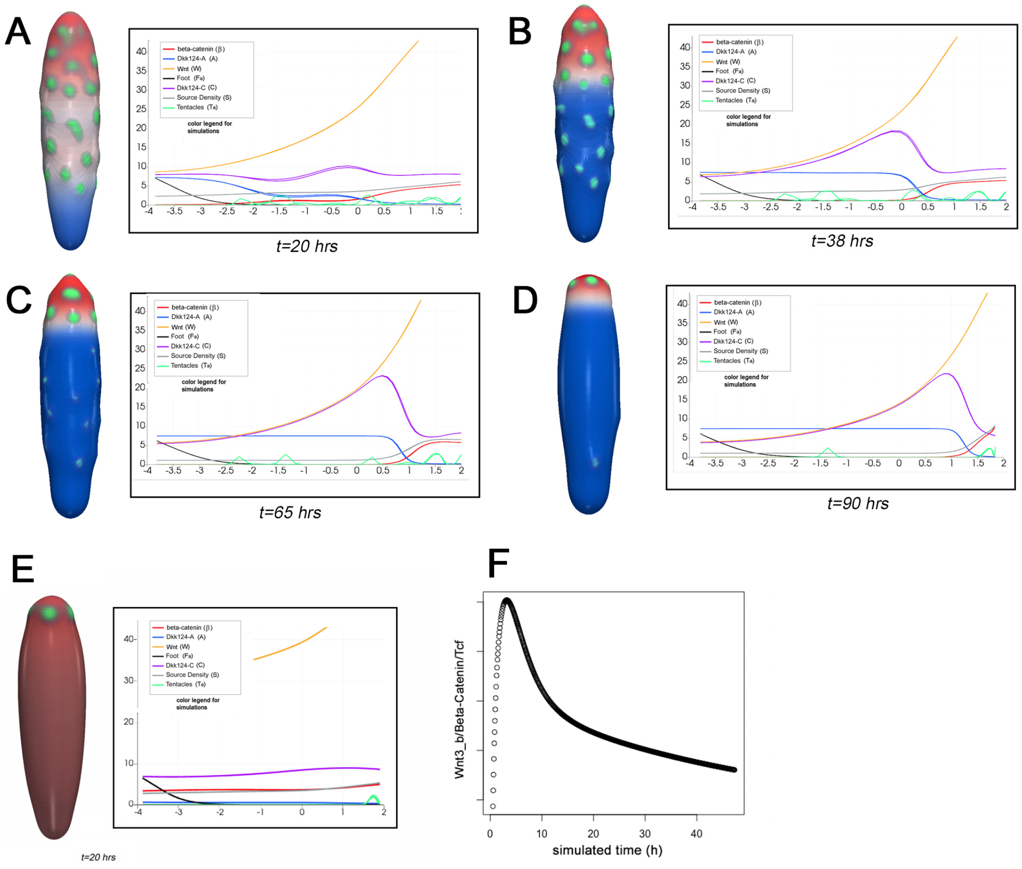

Fig H. (A–D): 3D results and extracted 1D concentration profiles for different time points after simulated ALP treatment. Red colour represents the [β] complex, blue colour Dkk1/2/4-A and green colour tentacles. (E): Snapshot of a simulation without the Dkk1/2/4-A-based inhibition of the [β] complex. Observed patterns (e.g., [W] gradients) result from [S]-initial conditions only, and vanish over time. I.e., the mutual negative feedback loop is required for local self-activation. (F): Simulated temporal development of relative β-Catenin expression strength after head removal.

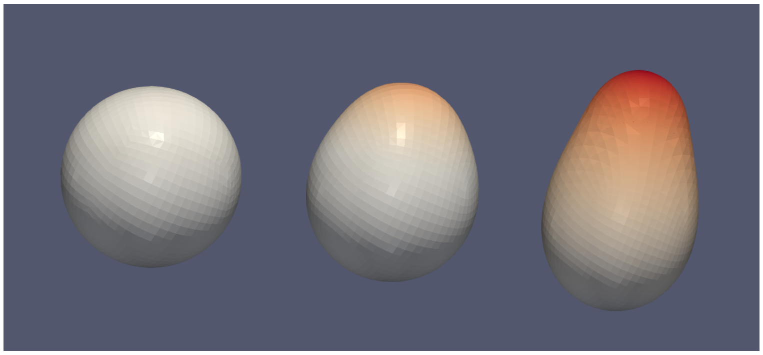

Fig I. Different stages of the 3D simulation of the *Hydra* aggregate system without foot and tentacle modules. The simulation corresponds to Fig 2D in the main text but excludes both auxiliary pattern-formation subsystems. The resulting *de novo* symmetry breaking and Wnt–Dkk distributions remain unchanged (apart from the absence of foot and tentacle structures), confirming that the foot and tentacle modules are purely morphological features and do not affect axis patterning.

To verify that the additional modules for foot and tentacle formation do not influence axis patterning, we repeated the 3D simulation of the *Hydra* aggregate system shown in Fig 2D of the main text with both modules removed. The resulting *de novo* symmetry breaking and steady-state axis pattern were indistinguishable from the full model. This confirms that the foot and tentacle systems are purely morphological extensions and have no feedback on the Wnt–Dkk mechanism responsible for axis formation (cf., Fig I).

Experimental and conceptual background of model components

The following section provides the biological and experimental background supporting the structure of the model equations presented in the main text. Each equation term corresponds to experimentally observed regulatory interactions or theoretical concepts that have been established in previous studies on *Hydra* axis formation and patterning.

Cell-local Wnt/β-catenin dynamics (Eq. 1): The variable $[\beta]$ represents intracellular canonical Wnt activity, summarizing the combined effects of β-catenin/Tcf signalling and expression of one or more canonical *HyWnt* genes. The production of $[\beta]$ is promoted by the source density ($[S]$), which defines the tissue's positional competence. This formulation reflects the experimental observation that tissues with higher head-forming capacity are more prone to activate Wnt/β-catenin signalling [13, 26–28]. The two Dkk proteins (HyDkk1/2/4-A and HyDkk1/2/4-C) act as inhibitors of this activity [29, 30], consistent with their mutually repressive interaction with canonical Wnt signalling. The remaining denominator term introduces a natural saturation of production, preventing unbounded activation, while the final term in Eq. (1) accounts for degradation or turnover of Wnt-related activity. Together, these terms reproduce the experimentally observed localization of strong Wnt activity at the apical tip and its suppression in the body column [11, 17, 28, 31–33].

Dkk1/2/4-A dynamics (Eq. 2): HyDkk1/2/4-A ($[A]$) acts as a short-range inhibitor of canonical Wnt signalling. Its expression is repressed by local Wnt activity ($[\beta]$), consistent with alsterpaullone (ALP) treatment experiments showing that *HyDkk1/2/4-A* expression is negatively regulated by Wnt/β-catenin activity and that its removal leads to ectopic *HyWnt3*-expressing clusters along the body column [29]. The inhibitory influence of $[A]$ on Wnt signalling has been confirmed both in *Hydra* and in *Xenopus* assays [29], and its knockdown combined with ALP treatment induces ectopic axis formation [34]. Eq. (2) therefore includes basal production repressed by $[\beta]$, small diffusion to mediate short-range inhibition, and a degradation term for turnover. Together, these interactions

capture the experimentally observed mutual inhibition between *HyDkk1/2/4-A* and canonical Wnt signalling.

Diffusible Wnt dynamics (Eq. 3): The diffusible Wnt variable, $[W]$, represents secreted Wnt ligands that spread over several cell diameters and mediate longer-range feedback. Its production depends on both $[S]$ and $[\beta]$, expressed as the multiplicative term $[S] \times [\beta]$. This coupling reflects the biological requirement that Wnt transcription and secretion occur only where tissue is both competent (high $[S]$) and actively signalling via β-catenin/Tcf (high $[\beta]$). β-Catenin/Tcf alone regulates thousands of genes [35] and is therefore not sufficient to specify Wnt gene expression or secretion. Additional slower regulatory layers—such as chromatin accessibility, stable cellular states, or other long-term determinants of gene expression—likely define whether a region is permissive for head-related gene expression. The $[S]$ field can thus be interpreted as a coarse-grained representation of such permissive conditions, while $[\beta]$ provides the immediate transcriptional drive. Only where both factors coincide can secreted Wnts be produced, ensuring robust and spatially confined activation of the head organizer. The diffusion of $[W]$ along the epithelial surface represents the lateral spread of secreted molecules, enabling intercellular coupling across the tissue. While studies in other organisms indeed suggest such diffusion [36–38], direct evidence in *Hydra* is still lacking. However, simulation results are robust over a broad range of diffusion constants (see Fig G A–D), and the qualitative patterning behaviour does not depend critically on these values. Finally, the last term in Eq. (3) accounts for degradation or clearance of secreted Wnt molecules.

Dkk1/2/4-C dynamics (Eq. 4): HyDkk1/2/4-C ($[C]$) represents a second inhibitory component with distinct regulation from $[A]$. Its production is positively influenced by diffusible Wnt ($[W]$) and negatively regulated by local Wnt activity ($[\beta]$), consistent with co-expression and LiCl treatment experiments [30]. This dual control ensures that $[C]$ is activated by long-range Wnt signals but suppressed within the head organizer itself, thereby restricting the spatial domain of Wnt activity. The broader spatial expression domain of *HyDkk1/2/4-C* compared to *HyWnt3*, as well as its characteristic ring-like pattern around the head region, suggests that activation and inhibition act on different spatial scales [18, 30]. This supports the inclusion of long-range activation by secreted Wnt ligands in Eq. (4). Removal of *HyDkk1/2/4-C*-expressing cells leads to extension of the *HyWnt3* domain into the head region [30], confirming its inhibitory role. Eq. (4) also includes small diffusion and degradation terms, reflecting the limited range and turnover of this inhibitor.

Source density dynamics (Eq. 5): The source density ($[S]$) captures a slowly varying, long-term field that encodes positional information along the oral–aboral axis of *Hydra*. This variable corresponds to the experimentally observed "head-forming capacity" or positional competence, which persists for several days after grafting or regeneration and depends on canonical Wnt/β-catenin activity [13, 26–28, 31]. Eq. (5) combines diffusion, Wnt-dependent production, and slow decay. Diffusion reflects the gradual redistribution of competence across the tissue, the production term represents induction of long-term competence by sustained Wnt/β-catenin activity [39], and the decay term models its relaxation back to baseline levels. Conceptually, $[S]$ can be interpreted as an emergent property arising from slow molecular, cellular, or possibly epigenetic mechanisms that determine which regions are permissive for head-related gene expression and organizer formation. This abstraction allows the model to capture the experimentally observed stability and spatial organization of the head-forming capacity without assuming a specific molecular identity.

In summary, each equation term is directly linked to experimental observations or well-established theoretical principles. The resulting system captures the interplay between short-term signalling (Wnt/β-catenin, Dkk feedback) and long-term positional memory (source density), providing a unified description of axis patterning in *Hydra*.

1D simulations of temporal aspects of head inhibition

Classical *Hydra* transplantation and fusion-type experiments suggested that an existing head can exert an inhibitory influence on subsequent head formation over extended tissue regions, and that this inhibitory effect may become established over time [40, 41]. To examine whether the MI model can reproduce this qualitative behaviour, we performed an additional set of simulations in the one-dimensional setting using initial conditions that mimic serially joined Hydra tubes with and without a head at one terminal end. Since the aim of this analysis was to visualise temporal differences in the emergence of secondary head-related peaks, we increased τ_5 in order to slow down the evolution of the source density and thereby make the transient dynamics more clearly observable. However, we want to point out that changes in τ values do not affect stationary solutions, thus all other presented simulation results of the 1D model are not dependent on this rescaling of τ_5.

The simulations show that in the absence of a terminal head, multiple secondary peaks emerge at approximately similar times along the joined construct (Fig J, upper row). In contrast, when a head is present at one end, peak formation in its vicinity is relatively suppressed or delayed, whereas a more distant secondary peak rises more strongly (Fig J, lower row). Thus, the model reproduces the qualitative expectation that a pre-existing organiser can bias the timing and relative strength of secondary head formation in extended tissue constructs. We interpret this result as consistent with the classical view that head inhibition in *Hydra* is both spatially extended and temporally developing [40, 41]. At the same time, this simulation is not intended as a detailed reconstruction of the historical experimental geometry, but rather as a focused test of the corresponding qualitative dynamical principle within the MI framework.

1D simulations of repeated ring grafts

To address the classical tandem graft experiments of Ando and Sawada, we performed an additional set of one-dimensional simulations mimicking extended chains of identical *Hydra* tissue pieces assembled from the same axial position [42]. The aim of this analysis was not to reproduce the full head-foot-bud system in quantitative detail, but to test whether the MI model predicts qualitatively different head-forming outcomes depending on the axial origin of the repeated pieces. To this end, we constructed extended initial source-density profiles by concatenating identical ring-like segments extracted from the fitted stationary pattern and added local perturbations of $[\beta]$ at the junctions to mimic wound-induced activation.

The simulations show a clear dependence of the number of resulting heads on the axial origin of the repeated pieces (Fig K). For chains assembled from mid-body pieces, only a small number of separated head-related peaks emerges, whereas chains assembled from pieces taken closer to the head region give rise to multiple peaks distributed along the construct. Thus, the model qualitatively reproduces the central observation that extended grafts composed of tissue with identical axial origin can generate markedly different patterning outcomes depending on their original position along the body axis [42].

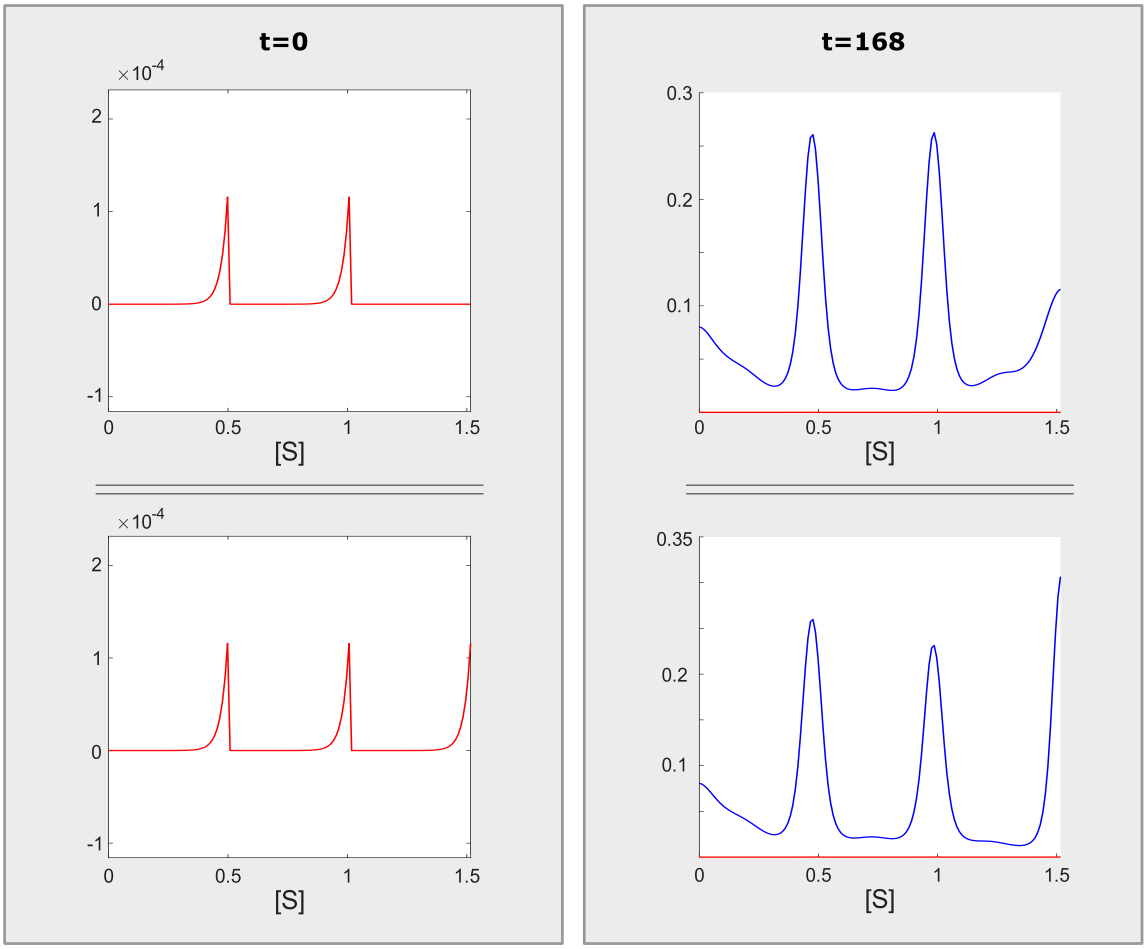

Fig J. Numerical simulation of the evolution of joined Hydra tubes using the one-dimensional model (S2). Parameter values are given in Table A, except for $\tau_5 = 100$, which was increased to slow down the evolution of the source density. The initial data (left) are taken as small perturbations of the homogeneous steady state for all components except the source density. For the source density, initial concentration profiles are extracted from the pattern shown in Fig B and connected in such a way as to mimic the situation with a head at the right end of the tube (lower row) and without a head (upper row). Blue curves show the evolving concentration profiles, while red curves denote the initial concentration values.

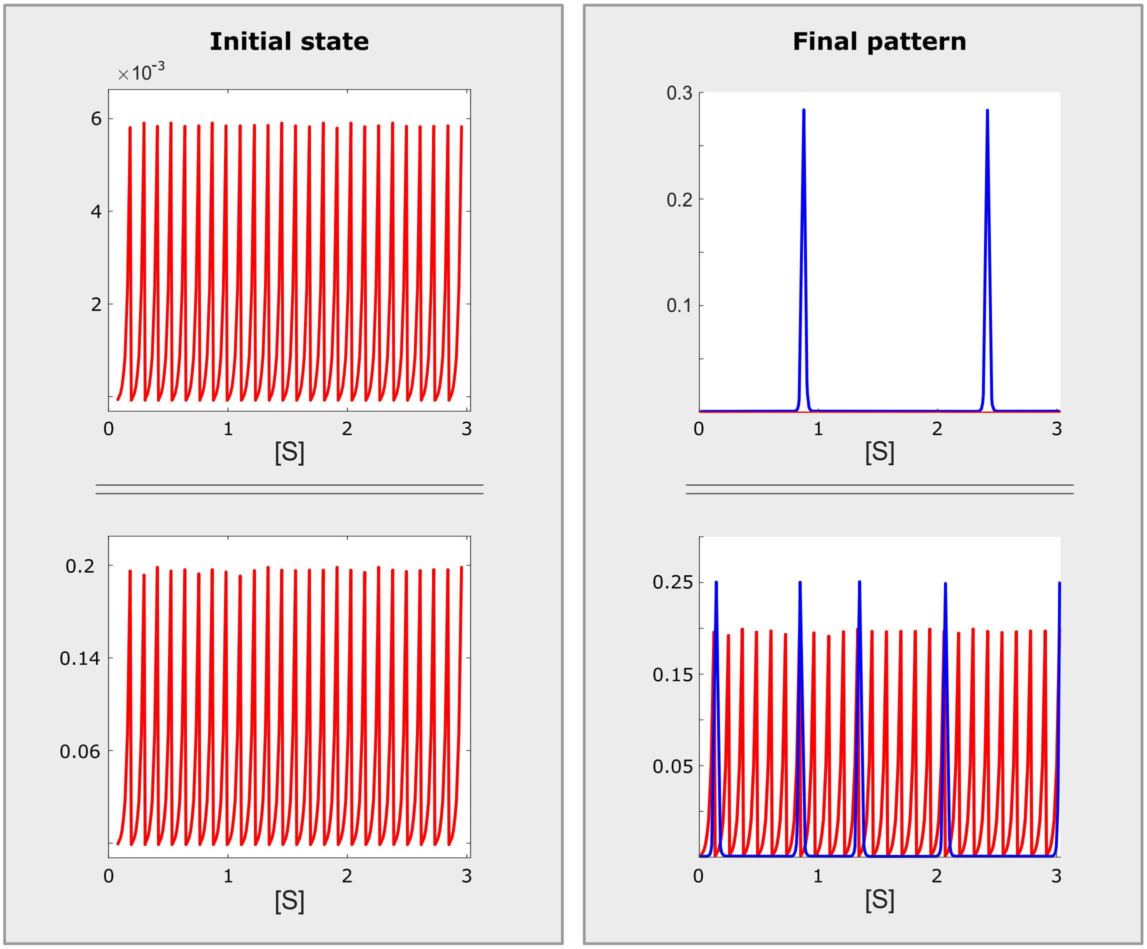

Fig K. Numerical simulation of the evolution of joined Hydra pieces (rings) [42] using the one-dimensional model (S2). Parameter values are taken from Table A, except for $\tau_5 = 5$, which is increased to slow down the evolution of the source density. Initial source-density profiles are extracted from the pattern shown in Fig B and concatenated to mimic a chain of pieces. All other components are initialised as small fluctuations around zero. To simulate the wound response, perturbations of $[\beta]$ concentrations are added at the junction sites. The spatial domain consists of 25 pieces, each representing approximately one-eighth of the body column. The upper row shows simulations for grafts composed of mid-body pieces, whereas the lower row corresponds to grafts assembled from pieces just below the head. Red curves denote the initial concentration profiles, and blue curves show the final profiles.

References

1. Yudovich VI. The onset of auto-oscillations in a fluid. Applied Mathematics and Mechanics. 1971;35(4):587–603.

2. Yudovich VI. Investigation of auto-oscillations of a continuous medium, occuring at loss of stability of a stationary mode. Applied Mathematics and Mechanics. 1972;36(3):587–603.

3. Mercker M, Marciniak-Czochra A, Richter T, Hartmann D. Modeling and computing of deformation dynamics of inhomogeneous biological surfaces. SIAM Journal on Applied Mathematics. 2013;73(5):1768–1792.

4. Mercker M, Hartmann D, Marciniak-Czochra A. A mechanochemical model for embryonic pattern formation: coupling tissue mechanics and morphogen expression. PLoS One. 2013;8(12):e82617. doi:10.1371/journal.pone.0082617.

5. Mercker M, Köthe A, Marciniak-Czochra A. Mechanochemical symmetry breaking in Hydra aggregates. Biophys J. 2015;108(9):2396–407. doi:10.1016/j.bpj.2015.03.033.

6. Helfrich W. Elastic properties of lipid bilayers: theory and possible experiments. Z Naturforsch [C]. 1973;28:693–703.

7. Philipp I, Aufschnaiter R, Özbek S, Pontasch S, Jenewein M, Watanabe H, et al. Wnt/beta-catenin and noncanonical Wnt signaling interact in tissue evagination in the simple eumetazoan Hydra. Proc Natl Acad Sci U S A. 2009;106(11):4290–5. doi:10.1073/pnas.0812847106.

8. Aufschnaiter R, Wedlich-Söldner R, Zhang X, Hobmayer B. Apical and basal epitheliomuscular F-actin dynamics during bud evagination. Biol Open. 2017;6:1137–48. doi:10.1242/bio.022723.

9. Amimoto Y, Kodama R, Kobayakawa Y. Foot formation in Hydra: a novel gene, anklet, is involved in basal disk formation. Mechanisms of development. 2006;123:352–361. doi:10.1016/j.mod.2006.03.002.

10. Technau U, Holstein TW. Head formation in Hydra is different at apical and basal levels. Development. 1995;121(5):1273–82.

11. Broun M, Gee L, Reinhardt B, Bode HR. Formation of the head organizer in Hydra involves the canonical Wnt pathway. Development. 2005;132(12):2907–16. doi:10.1242/dev.01848.

12. Smith KM, Gee L, Bode HR. HyAlx, an aristaless-related gene, is involved in tentacle formation in hydra. Development. 2000;127(22):4743–52.

13. Meinhardt H. Modeling pattern formation in hydra: A route to understanding essential steps in development. Int J Dev Biol. 2012;56(6-8):447–62. doi:10.1387/ijdb.113483hm.

14. Meinhardt H. A model for pattern formation of hypostome, tentacles, and foot in hydra: how to form structures close to each other, how to form them at a distance. Dev Biol. 1993;157(2):321–33.

15. Meinhardt H. Turing's theory of morphogenesis of 1952 and the subsequent discovery of the crucial role of local self-enhancement and long-range inhibition. Interface Focus. 2012;2:407–16. doi:10.1098/rsfs.2011.0097.

16. Becker R, Braack M, Dunne T, Meidner D, Richter T, Vexler B. Gascoigne 3D- a finite element toolbox (http://www.gascoigne.uni-hd.de); 2005.

17. Bode HR. Head regeneration in Hydra. Dev Dyn. 2003;226:225–36. doi:10.1002/dvdy.10225.

18. Mercker M, Lengfeld T, Höger S, Tursch A, Lommel M, Holstein TW, et al. Two separate but interconnected pattern formation systems are required to control body-axis and head-organiser formation in *Hydra*. BioRxiv. 2024; p. doi: https://doi.org/10.1101/2021.02.05.429954.

19. Livshits A, Shani-Zerbib L, Maroudas-Sacks Y, Braun E, Keren K. Structural Inheritance of the Actin Cytoskeletal Organization Determines the Body Axis in Regenerating Hydra. Cell Rep. 2017;18:1410–21.

20. Livshits A, Garion L, Maroudas-Sacks Y, Shani-Zerbib L, Keren K, Braun E. Plasticity of body axis polarity in Hydra regeneration under constraints. Scientific Reports. 2022;12 (13368).

21. Veschgini M, Petersen HO, Abuillan W, Rossetti FF, Özbek S, Burghammer M, et al. Spatio-temporal elasticity patterns in extracellular matrix during Hydra morphogenesis. bioRxiv. 2017;doi:https://doi.org/10.1101/214718.

22. Veschgini M, Suzuki R, Kling S, Petersen HO, Bergheim BG, Abuillan W, et al. Wnt/β-catenin signaling induces axial elasticity patterns of Hydra extracellular matrix. iScience. 2020;26 (4):10.1016/j.isci.2023.106416.

23. Ferenc J, Papasaikas P, Ferralli J, Nakamura Y, Smallwood S, Tsiairis CD. Mechanical oscillations orchestrate axial patterning through Wnt activation in Hydra. Science Advances. 2021;7 (50):eabj6897.

24. Weevers SL, Falconer AD, Mercker M, Sadeghi H, Rozema D, Ferenc J, et al. Mechanochemical Patterning Localizes the Organizer of a Luminal Epithelium. Science Advances. 2025;11(26):eadu2286. doi:10.1126/sciadv.adu2286.

25. Braun E, Ori H. Electric-induced reversal of morphogenesis in Hydra regeneration. Bioph J. 2019;117(8):1514–1523.

26. MacWilliams HK. Hydra transplantation phenomena and the mechanism of Hydra head regeneration. II. Properties of the head activation. Dev Biol. 1983;96:239–57.

27. Bode H. Axis formation in Hydra. Annu Rev Genet. 2011;45:105–17. doi:10.1146/annurev-genet-102209-163540.

28. Shimizu H. Transplantation analysis of developmental mechanisms in Hydra. Int J Dev Biol. 2012;56(6-7-8):463–72.

29. Guder C, Pinho S, Nacak TG, Schmidt HA, Hobmayer B, Niehrs C, et al. An ancient Wnt-Dickkopf antagonism in Hydra. Development. 2006;133:901–11. doi:10.1242/dev.02265.

30. Augustin R, Franke A, Khalturin K, Kiko R, Siebert S, Hemmrich G, et al. Dickkopf related genes are components of the positional value gradient in Hydra. Dev Biol. 2006;296:62–70. doi:10.1016/j.ydbio.2006.04.003.

31. Gee L, Hartig J, Law L, Wittlieb J, Khalturin K, Bosch T. Beta-catenin plays a central role in setting up the head organiser in hydra. Dev Biol. 2010;340:226–24.

32. Lengfeld T, Watanabe H, Simakov O, Lindgens D, Gee L, Law L, et al. Multiple Wnts are involved in Hydra organizer formation and regeneration. Dev Biol. 2009;330(1):186–99. doi:10.1016/j.ydbio.2009.02.004.

33. Hobmayer B, Rentzsch F, Kuhn K, Happel CM, von Laue CC, Snyder P, et al. Wnt signalling molecules act in axis formation in the diploblastic metazoan *Hydra*. Nature. 2000;407(6801):186–9. doi:10.1038/35025063.

34. Ziegler B, Yiallouros I, Trageser B, Kumar S, Mercker M, Kling S, et al. A Wnt-specific astacin proteinase controls head formation in Hydra. BMC Biol. 2021;19(1):doi: 10.1186/s12915–021–01046–9.

35. Reddy PC, Gungi A, Ubhe S, Galande S. Epigenomic landscape of enhancer elements during Hydra head organizer formation. Epigen & Chrom. 2020;13(43):1–16.

36. Mii Y, Nakazato K, Pack CG, Ikeda T, Sako Y, Mochizuki A, et al. Quantitative Analyses Reveal Extracellular Dynamics of Wnt Ligands in Xenopus Embryos;10:e55108. doi:10.7554/eLife.55108.

37. Pani AM, Goldstein B. Direct Visualization of a Native Wnt in Vivo Reveals That a Long-Range Wnt Gradient Forms by Extracellular Dispersal;7:e38325. doi:10.7554/eLife.38325.

38. Recouvreux P, Pai P, Torro R, Ludányi M, Mélénec P, Boughzala M, et al.. Establishment of Wnt Ligand-Receptor Organization and Cell Polarity in the C. Elegans Embryo;. Available from: https://www.biorxiv.org/content/10.1101/2023.01.17.524363v1.

39. Tursch A, Bartsch N, Mercker M, Schlüter J, Lommel M, Özbek AMCA, et al. Injury-induced MAPK activation triggers body axis formation in Hydra by default Wnt signaling. PNAS. 2022;119(35):e2204122119.

40. Tardent P. Axiale Verteilungs-Gradienten der interstitiellen Zellen bei Hydra und Tubularia und ihre Bedeutung für die Regeneration. Wilhelm Roux' Archiv für Entwicklungsmechanik der Organismen. 1954;146(5–6):593–649. doi:10.1007/BF00576515.

41. Wilby OK, Webster G. Studies on the transmission of hypostome inhibition in hydra. JEEM. 1970;24:583–593.

42. Ando H, Sawada Y, Shimizu H, Sugiyama T. Pattern formation in hydra tissue without developmental gradients. Developmental Biology. 1989;133(2):405–414. doi:10.1016/0012-1606(89)90044-4.

S1 Appendix Legends

Table A in S1 Appendix. Parameter values of the one-dimensional model

Values of model parameters obtained by fitting the one-dimensional model (S2) to the pattern data obtained from numerical integration of the pseudo-3D model.

Table B in S1 Appendix. Baseline parameter values used for the pseudo-3D simulations

Baseline parameter values used for the pseudo-3D simulations, including the mutual inhibition (MI) model and the auxiliary tentacle and foot modules.

Fig A in S1 Appendix. Intersections of functions $f_1(w)$ and $f_2(w)$

Intersections of the functions $f_1(w)$ and $f_2(w)$ for different values of the model parameters ζ_i, $i = 1, \ldots, 6$. The value of ζ_1 is indicated in each panel, while all other parameters are fixed to the values given in Table A.

Fig B in S1 Appendix. Comparison of one-dimensional steady-state patterns with pseudo-3D simulation data

Steady-state pattern obtained from the numerical simulation of the one-dimensional model (S2) with the parameter values from Table A, compared with concentration profiles from the pseudo-3D simulations averaged in the direction perpendicular to the body axis. Both concentration profiles are scaled to the interval $[0, 1]$ using min–max normalisation.

Fig C in S1 Appendix. Examples of pattern formation for different initial conditions

Three examples of pattern formation in the one-dimensional model. Parameter values used in the simulations are given in Table A. Initial data are taken as small perturbations of the homogeneous steady state. Blue curves show the final concentration profiles, while red curves denote the initial concentration values.

Fig D in S1 Appendix. Semi-analytical bifurcation analysis of the one-dimensional system

Results of the semi-analytical bifurcation analysis of the one-dimensional system. (A) Real parts of the eigenvalues of the linear operator $L_N(\nu_{\text{cr}})$. Real eigenvalues are shown in blue, while the real parts of complex eigenvalues are shown in green. The critical eigenvalue is highlighted in red. (B) Unstable modes as functions of the diffusion coefficient of $[W]$. (C) Asymptotics of the secondary stationary solution for the first component, evaluated at $\epsilon = 0.01$ (blue), compared with the numerical solution computed with the same parameter values (red). The green dashed line represents the respective component of the spatially homogeneous steady state w_1. Initial conditions for the simulation are small random perturbations of the stationary state.

Fig E in S1 Appendix. Pattern of the model variant with feedback in degradation terms

Example pattern obtained by numerically integrating system (S34)–(S38), in which regulatory feedback is introduced via modified degradation terms. Initial data are taken as small perturbations of the homogeneous steady state w_1. Blue curves show the final concentration profiles, while red curves denote the initial concentration values.

Fig F in S1 Appendix. Pattern of the model variant with nonlinear reaction terms

Example pattern obtained by numerically integrating system (S39)–(S43), in which selected reaction terms are replaced by higher-order nonlinear terms. Initial data are taken as small perturbations of the homogeneous steady state w_1. Blue curves show the final concentration profiles, while red curves denote the initial concentration values.

Fig G in S1 Appendix. Parameter sensitivity analysis and model variants

(A–D) Stable pseudo-3D patterns and extracted one-dimensional concentration profiles for models with different diffusion rates of the $[\beta]$ complex (B) or of $[W]$ (C–D), compared with the unperturbed system (A). (E–H) Simulated rescaled distribution of Dkk1/2/4-C (E–F) and $[\beta]$ (G–H) expression for the undisturbed system (E,G) and for a system in which no $[\beta]$-based inhibition of Dkk1/2/4-C is included (F,H). In the latter case, the expression patterns resemble the classical activator–inhibitor model. (I–J) Relative distribution of simulated Dkk1/2/4-A (blue), $[\beta]$ (red), and tentacle (green) expression for the undisturbed system (I) and for a system without constant Dkk1/2/4-A expression

but with $[S]$-induced expression instead (J). (K–M) Concentration profiles of the unperturbed system (K) compared with systems with equal Dkk1/2/4-A and Dkk1/2/4-C diffusion rates. In (L), $a_2 = a_4 = 1 \times 10^{-6}$; in (M), $a_2 = a_4 = 1 \times 10^{-7}$.

Fig H in S1 Appendix. ALP-treatment simulations

(A–D) Pseudo-3D results and extracted one-dimensional concentration profiles for different time points after simulated ALP treatment. Red colour represents the $[\beta]$ complex, blue colour Dkk1/2/4-A, and green colour tentacles. (E) Snapshot of a simulation without Dkk1/2/4-A-based inhibition of the $[\beta]$ complex. The observed patterns, such as $[W]$ gradients, result from $[S]$ initial conditions only and vanish over time, indicating that the mutual negative feedback loop is required for local self-activation. (F) Simulated temporal development of relative β-catenin expression strength after head removal.

Fig I in S1 Appendix. Symmetry breaking in aggregate simulations without auxiliary modules

Different stages of the pseudo-3D simulation of the *Hydra* aggregate system without foot and tentacle modules. The simulation corresponds to Fig 2D in the main text but excludes both auxiliary pattern-formation subsystems. The resulting de novo symmetry breaking and Wnt–Dkk distributions remain unchanged, apart from the absence of foot and tentacle structures, confirming that the foot and tentacle modules are purely morphological features and do not affect axis patterning.

Fig J in S1 Appendix. Simulations of temporal aspects of head inhibition

Numerical simulation of the evolution of joined *Hydra* tubes using the one-dimensional model (S2). Parameter values are given in Table A, except for $\tau_5 = 100$, which was increased to slow down the evolution of the source density. The initial data (left) are taken as small perturbations of the homogeneous steady state for all components except the source density. For the source density, initial concentration profiles are extracted from the pattern shown in Fig B and connected to mimic the situation without a head (upper row) and with a head at the right end of the tube (lower row). Blue curves show the evolving concentration profiles, while red curves denote the initial concentration values.

Fig K in S1 Appendix. Simulations of head formation frequency in repeated ring grafts

Numerical simulation of the evolution of joined *Hydra* pieces (rings) using the one-dimensional model (S2). Parameter values are taken from Table A, except for $\tau_5 = 5$, which was increased to slow down the evolution of the source density. Initial source-density profiles are extracted from the pattern shown in Fig B and concatenated to mimic a chain of pieces. All other components are initialised as small fluctuations around zero. To simulate the wound response, perturbations of $[\beta]$ concentrations are added at the junction sites. The spatial domain consists of 25 pieces, each representing approximately one-eighth of the body column. The upper row shows simulations for grafts composed of mid-body pieces, whereas the lower row corresponds to grafts assembled from pieces just below the head. Red curves denote the initial concentration profiles, and blue curves show the final profiles.
